# Negative pressure wound therapy versus usual care in patients with surgical wound healing by secondary intention in the UK (SWHSI-2): an open-label, multicentre, parallel-group, randomised controlled trial

**DOI:** 10.1016/S0140-6736(25)00143-6

**Published:** 2025-05-10

**Authors:** Catherine Arundel, Laura Mandefield, Caroline Fairhurst, Kalpita Baird, Athanasios Gkekas, Pedro Saramago, Ian Chetter, SWHSI-2 Trial Investigators, Belen Corbacho Martin, Catherine E Hewitt, Andrew Mott, Samantha Swan, David J Torgerson, Jacqueline Wilkinson, Sabeen Zahra, Jane Blazeby, Rhiannon Macefield, Stephen Dixon, Josie Hatfield, Angela Oswald, Jo Dumville, Matthew Lee, Thomas Pinkney, Nikki Stubbs, Lyn Wilson, Annie Clothier, David C Bosanquet, Melissa Blow, Claire Price, Jackie Todd, Tracey Munro, Woolagasen Pillay, Aniket Pradhan, Andrew Garnham, Michael Wall, Katarzyna Powezka, Arooj Syed, David Gerrard, Andrea Croucher, Nansi Hadjievangelou, Anna Firth, Tracey Roe, George Smith, Colin Bicknell, Caitlin Carr, Esther Negbenose, Lawrence. Tarusan, Alex Vesey, Debbie Wilson, Dina Bell, Joanne Fletcher, Clare Greenwood, Tom Wallace, Srinivasa Vallabhaneni, Sophie Holder, Julie Williams, Steven Sim, Andrew L Tambyraja, Fiona Kerray, Aaron Ng, Martin Sylvester, Lynda Slater, S Tawqeer Rashid, April Palacios, Kornelia Feld, Sandip Nandhra, Gerard Stansby, Noala Parr, Louise Jones, Jeanette Milne, Caroline Stubbs, Robert Hinchliffe, Christopher Twine, Georgios A Antoniou, Carolyn Corbett, Sheila Munt, Sarah Warran, Rachel Fletcher, Wissam Al-Jundi, Mandy Burrows, Philip Stather, Rachel Barnes, Tania Woodrow, Benita Adams, Obiekezie Agu, Yvonne Gleeson, Rovan DSouza, Leanna Erete, Steven Jones, Clare Checketts, Dani Bajic, Robert Matravers, Ian Loftus, James Budge, Bilal Azhar, Rebecca Hancox, Cheri Pearce, Nigel Suggett, Arlo Whitehouse, Maciej Juszczak, Ganesh Kuhan, Sivaram Premnath, Nikesh Dattani, Vanessa Hollings, Farah Khasawneh, Julien AlShakarchi, Emily Packer

**Affiliations:** a York Trials Unit, University of York, York, UK; b Centre for Health Economics and University of York, York, UK; c Faculty of Health Sciences, University of Hull, Hull, UK; d Hull York Medical School, Hull, UK; e Hull University Teaching Hospitals NHS Trust, Hull, UK

## Abstract

**Background:**

Surgical wound healing by secondary intention (SWHSI) presents a substantial management and financial challenge. Negative pressure wound therapy (NPWT) has increasingly been used as a treatment despite an absence of comparative evidence of effectiveness. We evaluated the effectiveness of NPWT compared with usual care for improving time to wound healing in patients with an SWHSI.

**Methods:**

We did a pragmatic, open-label, multicentre, parallel-group, randomised controlled trial in 29 UK National Health Service Trusts. Participants aged 16 years or older with an SWHSI appropriate for both study treatments (NPWT or usual care) were randomly assigned (1:1) by a centralised web-based system. Randomisation was stratified by wound location, wound area, and study centre. Participants were followed up for 12 months. Participants and clinical and research teams could not be masked to treatment. Assessors masked to treatment reviewed wound photography to verify the outcome. The primary outcome was time to wound healing (days from randomisation to complete epithelial cover), analysed via intention to treat using Kaplan–Meier survival curves and a proportional hazards Cox regression model. The trial was registered with ISRCTN, ISRCTN26277546.

**Findings:**

Between May 15, 2019, and Jan 13, 2023, 686 participants with an SWHSI were randomly assigned to receive NPWT (n=349) or usual care (n=337). All participants were included in the primary analysis. Most participants were diabetic (n=549, 80·0%) and had a single SWHSI (n=622, 90·7%), located on the foot or leg (n=620, 90·4%), arising after vascular surgery (n=619, 90·2%). There was no clear evidence that NPWT reduced the time to wound healing compared with usual care (hazard ratio 1·08 [95% CI 0·88–1·32], p=0·47). There were 448 adverse events, of which 14 were serious (nine participants in the NPWT group and five participants in the usual care group); 124 were deemed potentially related to treatment. NPWT was found not to be cost-effective compared with usual care.

**Interpretation:**

In patients with a lower limb SWHSI, including those with complications of diabetes, there is no clear evidence that NPWT reduced the time to wound healing compared with standard dressings. These findings do not support the use of NPWT to augment SWHSI healing.

**Funding:**

National Institute for Health Research Health Technology Assessment Programme.

## Introduction

Healing by secondary intention refers to a strategy to heal wounds by leaving skin edges unopposed. This method requires the growth of granulation tissue from the base of the wound and might be preferred in areas where there is contamination, or inability to achieve primary skin cover.

Available prevalence data for wound healing by secondary intention, derived from a UK survey, indicates these wounds are common, with an estimated prevalence of 4·1 per 10 000 population in the UK.^1^ Median time to healing for these wounds is prolonged at 86 days (95% CI 75–130). There is, however, variation in healing times for different types of surgical wound healing by secondary intention (SWHSI), with those located on the foot or leg having a median time to healing that is double those located elsewhere on the body.^2^ Wound infection (32·1%), hospital readmission (24·7%), and further surgical procedures (16·8%) are common in patients with an SWHSI,^2^ which substantially impacts health-related quality of life.^3^, ^4^ These wounds are costly to health services, with the mean cost of an SWHSI estimated to be between £1500 and £2400 per month depending on the treatments used.^5^

A range of wound dressings are used in the management of SWHSI, ranging from simple dressings such as gauze, to more advanced dressings such as hydrofibre or alginate.^6^ Frequent changes to these dressings are required. An alternative approach, negative pressure wound therapy (NPWT), uses a controlled vacuum to remove wound fluid.^7^ It is claimed that the vacuum creates an environment more conducive to wound healing by removing infective materials and exudate, reducing oedema, and promoting perfusion and granulation.^8^

**Research in context**

**Evidence before this study**

Healing by secondary intention refers to a strategy to heal wounds by leaving skin edges unopposed. This method requires the growth of granulation tissue from the base of the wound and might be preferred in areas where there is contamination, or inability to achieve primary skin cover. Healing by secondary intention is common, with an estimated prevalence of 4·1 per 10 000 population. Median time to healing for these wounds is prolonged (86 days [95% CI 75–130]), with wounds located on the foot or leg taking more than double the time to heal of those located elsewhere on the body. Additional treatments are often required, resulting in high health-care costs, and can affect patient quality of life. Alongside conventional wound dressings, an alternative called negative pressure wound therapy (NPWT) can be used as a dressing. This approach uses a controlled vacuum to remove wound fluid, which manufacturers claim results in a conducive wound healing environment. The use of NPWT for surgical wound healing by secondary intention (SWHSI) has increased rapidly in recent years, with a 23% increase in use reported between 2012 and 2014; however, there is an absence of robust supporting evidence regarding its clinical and cost-effectiveness. A Cochrane systematic review of NPWT for SWHSI identified two small, low-quality randomised controlled trials and recommended caution when interpreting the findings and that further high-quality randomised controlled trials be conducted.

**Added value of this study**

In this pragmatic, open-label, multicentre, parallel-group, randomised, controlled trial (SWHSI-2) we aimed to undertake the first robust evaluation of the clinical and cost-effectiveness of NPWT compared with usual care (no NPWT) in treating SWHSI. We found no evidence that NPWT reduced the time to wound healing compared with standard dressings in a population with predominantly lower limb wounds, including those with complications of diabetes. The time taken for the wounds to heal did not differ significantly between groups and there were no statistically significant differences between groups in the odds of relevant clinical secondary outcomes (readmission, reoperation, infections and antibiotic use, amputation, or death), patient-reported outcomes (Bluebelle Wound Healing Questionnaire and visual analogue scale pain scores), or masked outcome assessment using wound photographs.

After adjustment for baseline characteristics and EQ-5D-5L scores, NPWT was also found to increase patient quality of life and costs; however, these results were not statistically significant.

**Implications of all the available evidence**

Despite manufacturers' claims that NPWT promotes wound healing, the findings in our large study do not support this claim. NPWT does not confer an advantage in terms of time to healing or adverse events over standard care, particularly for patients with lower limb SWHSI, including those with complications of diabetes. This finding suggests that NPWT should not be considered as a first-line treatment for such patients in relation to wound healing and highlights the challenges that remain in wound care when new dressings are introduced without comparative evidence and the clear need to identify new candidate interventions for the healing of SWHSI.

The use of NPWT for SWHSI has increased rapidly in recent years, with a 23% increase in use reported between 2012 and 2014;^6^ however, there is absence of robust supporting evidence regarding its clinical effectiveness and cost-effectiveness. A Cochrane systematic review of NPWT for SWHSI identified two, small, low-quality randomised controlled trials,^9^, ^10^, ^11^, ^12^ and recommended caution when interpreting the findings and that further high-quality randomised controlled trials should be conducted.^7^, ^13^

The SWHSI-2 trial therefore sought to assess the clinical effectiveness and cost-effectiveness of NPWT compared with usual care (no NPWT) in treating SWHSI.

## Methods

### Study design and participants

In SWHSI-2, an open-label, multicentre, parallel-group, randomised controlled superiority trial, we compared the effects of NPWT with usual care on time to SWHSI healing (as days since randomisation). The trial was registered with ISRCTN, ISRCTN26277546. The protocol has previously been published^14^ and is included in the appendix (pp 1–44).

Recruiting sites comprised 28 secondary care and one community UK National Health Service Trusts. A list of participating centres is provided in the appendix (p 45). Eligible patients were aged 16 years or older with an acute SWHSI (<6 weeks from surgery resulting in an SWHSI or wound dehiscence) anywhere on the body, which was considered appropriate for NPWT treatment (ie, ≥80% viable tissue or thin layer of slough not requiring debridement). Patients were ineligible if they were malnourished,^15^, ^16^ or had a life expectancy of less than 6 months, active systemic infection, chronic or non-surgical wounds, risk of bleeding or inadequate haemostasis, contraindications to NPWT, or were participating in an ongoing wound care trial where the primary outcome had not been reached. The SWHSI was also ineligible if NPWT had already or previously been applied or if delayed primary closure was planned.

The Yorkshire and Humber Leeds East Research Ethics Committee and UK Health Research Authority granted ethical approval (reference 19/YH/0054). Eligible participants were required to give written informed consent.

### Randomisation and masking

Randomisation to NPWT or usual care (1:1) was stratified by wound location (foot and ankle, leg, abdomen, or other), wound area (<28 cm^2^ or ≥28 cm^2^, calculated as the measurement arising from the longest part of the wound multiplied by the measurement for the widest part of the wound), and study centre, using variable block sizes (two, four, six, and eight). The allocation sequence was generated independently by the trial statistician and was implemented using a bespoke, centralised, secure online randomisation service developed and hosted by York Trials Unit, University of York (York, UK). The clinical and research teams accessed the online system via individual log on, to complete randomisation as required.

Participants and the clinical and research teams were unmasked to treatment allocation. However, to minimise bias, masked assessment of wound healing photographs was completed by clinically experienced independent observers and analysis of this was included as a secondary outcome.^17^

### Procedures

After screening, eligible patients were approached for informed consent. Baseline questionnaires were then completed, and participants were randomly assigned (1:1) to intervention (NPWT) or control (usual care).

Participants in the intervention group were randomly assigned to be treated with NPWT applied to their wound in accordance with manufacturer guidance. A specific NPWT model was not prescribed; however, the device was required to be CE marked (ie, meeting legislative requirements for use in the UK and Europe) and to provide 60–150 mm Hg pressure. Given that there is no evidence to suggest an optimal duration of use or that any one pressure cycle (continuous or intermittent), dressing, or use of a liner (added wound contact layer to prevent dressing adherence) is beneficial to wound healing, the choice of these clinical parameters was left to the local clinical care team to determine on a per-patient basis.

Control group participants were randomly assigned to receive routine wound care dressings without NPWT. There is no evidence of any one dressing type or brand being more clinically effective or cost-effective;^18^ therefore, primary and secondary dressings, and frequency of change, were determined by the clinical care team.

Participants were followed up for 12 months, with weekly telephone contact by research nurses to collect the data on primary and clinical secondary outcomes and adverse events. When healing was reported and confirmed by a health-care professional as meeting the study definition (complete epithelial cover in absence of a scab), a further visit was completed (face to face) to obtain wound photographs. Where face-to-face follow-up was not possible, the visit was conducted by video or telephone call and participants were asked to provide a wound photograph to the central study team. Follow-up continued by telephone for a further 2 weeks to confirm continued wound healing. Study follow-up was ongoing at the time of the COVID-19 pandemic restrictions in the UK. From March, 2020, until the end of study follow-up (Jan 13, 2024), where necessary, weekly telephone calls were conducted by the central coordinating centre on behalf of site research nurses to ensure continued data collection during this period.

Data on gender or race were collected by the investigator via medical records or discussion with the patient as necessary. Epidemiological data, including patient demographics (eg, date of birth, gender, and ethnicity), comorbidities, smoking status, surgical procedure details (eg, urgency, contamination level, and type), and a wound photograph were collected at baseline. Patient-reported outcome measures included indications of wound infection via the Bluebelle Wound Healing Questionnaire (WHQ;^19^, ^20^ at baseline and 3-month follow-up), wound pain via the visual analogue scale (VAS, in which 0 indicated no pain and 100 indicated worst imaginable pain; at baseline and 3-month follow-up), health-related quality of life using the EQ-5D-5L^21^ (at baseline and 3-month, 6-month, and 12-month follow-up), and resource use (eg, wound-related National Health Service consultations, support, and out-of-pocket costs; at baseline and 3-month, 6-month, and 12-month follow-up) at baseline and at 3 months, 6 months, and 12 months after randomisation using postal questionnaires. Data collection timepoints for WHQ and VAS were revised in October, 2022, to baseline, 3-month follow-up, and post healing only to reduce participant burden, with the aim of improving questionnaire response rates. Outcome data were collected using bespoke case report forms developed for the study. These data were processed at the York Trials Unit, University of York, using a licensed, automated, electronic system (Teleform version 11.2), which allows data to be entered, checked, and validated. Procedural details pertaining to the processing of the data were documented in a study-specific data management plan.

### Outcomes

The primary outcome for this study was time to healing (unmasked) defined as complete epithelial cover in the absence of a scab in days from randomisation. Clinical secondary outcomes were wound infection (as determined by the treating health-care professional) and antibiotic treatment, hospital admission or discharge, current treatment and reasons for change (if applicable), reoperation, amputation, and death within 12 months after randomisation. Masked assessment of wound healing photographs and time to healing assessment was also assessed as a secondary outcome. Adverse events relating to the wound or study treatments were collected as a secondary outcome. Prespecified expected adverse events included minor wound infection, cellulitis, oedema, and maceration and retention of product in the wound (eg, wound filler embedding in granulated tissue).

### Statistical analysis

A conservative estimate of a 25% decrease in time to healing was used on the basis of available literature,^9^, ^10^, ^11^, ^22^ patient and public opinion, and cost-effectiveness modelling data,^5^ representing both a clinically meaningful difference (to patients) and a target difference (as derived from literature).

To detect a 25% reduction in median time to healing (from 86 days with usual care to 65 days with NPWT equating to a hazard ratio [HR] of 1·32 and a control group event proportion of 0·95), with 90% power, two-sided type I error rate of 5%, a 12-month follow-up period, and allowing for 20% attrition,^2^, ^10^, ^23^ 696 participants were required (348 per group).

Statistical analysis was conducted in R (version 4.4.0) and Stata (version 18) using two-sided significance tests at the 5% significance level, with parameter estimates presented with associated 95% CI and p values as appropriate. All analyses were done on the intention-to-treat population (all participants who were randomly assigned, analysed according to random allocation) unless stated otherwise. A statistical analysis plan was prepared before completion of data collection. An external Data Monitoring and Ethics Committee met annually to review blinded recruitment and baseline and safety data. There were no planned interim analyses or stopping rules for this trial.

The primary outcome (time to healing of the reference wound) was assessed using a Kaplan–Meier curve by group and the median time to healing and HR with 95% CIs was calculated using a proportional hazards Cox regression model in the coxme package.^24^ Baseline wound size (cm^2^), wound duration (wound start date to randomisation), and wound location were adjusted for as fixed effects, and centre as a shared frailty effect. Mean imputation was used to impute 19 (2·8%) missing baseline values for wound area (within the same stratification factor) and three (0·4%) for wound duration (overall mean). Time to healing was right-censored at the last timepoint at which the wound was known to still be unhealed (ie, the earliest of 12 months after randomisation, loss to follow-up, full withdrawal, death, or amputation of the SWHSI).

Sensitivity analyses were completed to account for death and amputation as competing risks (using Fine and Gray's proportional subhazards model), to correct for stratification errors and to adjust for baseline imbalances in smoking and alcohol consumption.^25^ The impact of compliance was assessed with a Complier Average Causal Effect analysis using an instrumental variable approach,^26^ and a prespecified subgroup analysis was undertaken by including an interaction term between previous SWHSI history and randomised group in the primary analysis model.

Antibiotic treatment, hospital admission or discharge, current treatment and reasons for change, reoperation, amputation, and death events were analysed using a mixed-effect logistic regression model adjusting for the same covariates. The masked wound healing assessments were also analysed using the same approach as for the primary outcome, with other wounds treated as healed in the primary analysis being censored at the date of healing.

WHQ scores (at 3 months, 6 months, and 12 months) and wound pain (VAS) were analysed using a covariance-pattern mixed model adjusting for baseline measure, wound size, wound duration, and wound location as fixed effects, and centre and participant as random effects.

Adverse events were summarised by trial group and overall. Further details regarding these analyses, where relevant, are available in the appendix (pp 46–52).

Following the UK National Institute for Health and Care Excellence guidelines,^27^ and under a National Health Service and Personal Social Services perspective, a within-trial economic analysis was performed using Stata (version 18) to compare NPWT and usual care for SWHSI based on SWHSI-2 trial intention-to-treat data. The EQ-5D-5L questionnaire was used to estimate health-related quality of life. Quality-adjusted life-years (QALYs) were estimated using the area under the curve approach. Data on trial interventions, medication, and primary and secondary health-care resource use collected within the trial were costed. The economic analysis was performed within the study's follow-up period of 12 months, hence costs and QALYs were not discounted.

Total QALYs and costs were aggregated and analysed for each treatment group with missing data imputed via multiple imputation by chained equations. Adjusted incremental QALYs and incremental costs were estimated via seemingly unrelated regressions that controlled for participants' baseline characteristics in both QALYs and total costs simultaneously, accounting for any potential correlation. A bootstrap approach was used to obtain 95% CIs. Incremental net monetary benefit at the £20 000 and £30 000 cost-effectiveness thresholds are presented, as well as the cost-effectiveness plane and acceptability curves. Sensitivity analyses were carried out to explore the robustness of the base-case cost-effectiveness results. Further details regarding these analyses, where relevant, are available in the appendix (pp 53–70).

### Role of the funding source

The funder of the study had no role in study design, data collection, data analysis, data interpretation, or writing of the report.

## Results

**Figure 1:**
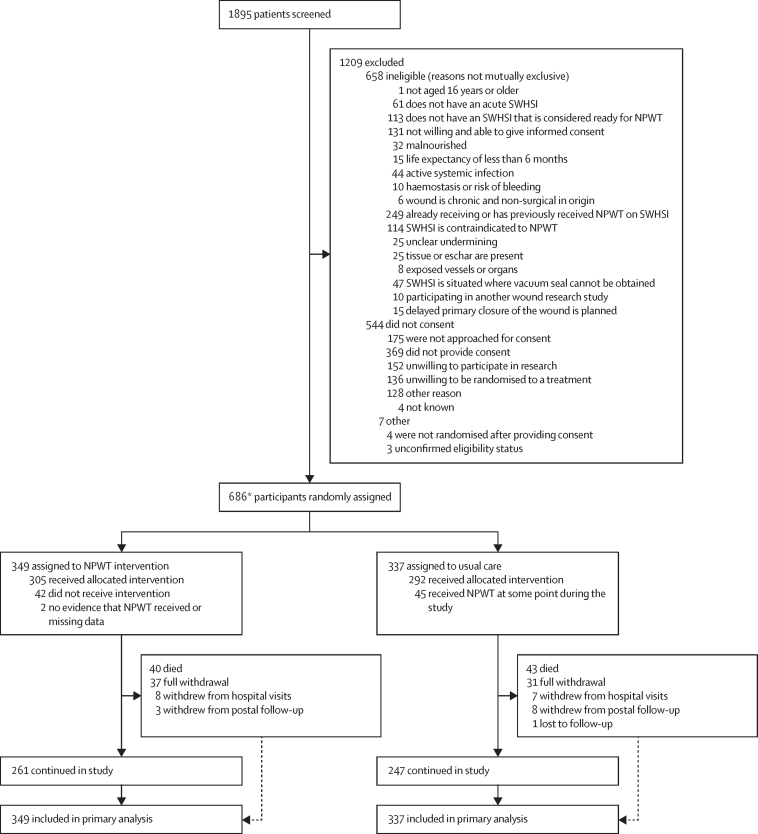
Trial profile NPWT=negative pressure wound therapy. SWHSI=surgical wound healing by secondary intention. *There were six system randomisation errors where the same participant was randomised multiple times. These were removed from any analysis, leaving 686 participants.

Between May 15, 2019, and Jan 13, 2023, 1895 patients with an SWHSI were screened and 686 (36·2%) were randomly assigned to NPWT (n=349) or usual care (n=337; figure 1). Participants were followed up for 12 months after randomisation, and the final follow-up was completed on Jan 13, 2024.

Baseline characteristics of study participants were considered balanced across groups for all categories, except for smoking status and alcohol consumption (table 1). The median participant age was 63 years (IQR 55–72), most participants were male (n=513, 74·8%), and of White ethnicity (n=630, 91·8%).

**Table 1:** Baseline characteristics for the intention-to-treat population by treatment group

			**NPWT (n=349)**	**Usual care (n=337)**
Age, years			62·00 (54·00–71·0)	64·00 (55·0–72·0)
Gender				
	Male		267 (76·5%)	246 (73·0%)
	Female		79 (22·6%)	91 (27·0%)
	Missing		3 (0·9%)	0
Ethnicity				
	White		317 (90·8%)	313 (92·9%)
	Asian or Asian British		14 (4·0%)	14 (4·2%)
	Black or Black British		11 (3·2%)	9 (2·7%)
	Other ethnicity		1 (0·3%)	0
	Missing		6 (1·7%)	1 (0·3%)
Smoking status				
	Never		121 (34·7%)	143 (42·4%)
	Current		66 (18·9%)	53 (15·7%)
	Previous		158 (45·3%)	140 (41·5%)
	Missing		4 (1·1%)	1 (0·3%)
Routine alcohol consumption*				
	Yes		125 (35·8%)	128 (38·0%)
	No		219 (62·8%)	205 (60·8%)
	Missing		6 (1·7%)	9 (2·7%)
Comorbidities				
	Cardiovascular disease (hypertension, myocardial infarction, angina, or heart failure)			
		Yes	218 (66·3%)	228 (71·9%)
		No	111 (31·8%)	89 (26·4%)
		Missing	20 (5·7%)	20 (5·9%)
	Peripheral vascular disease			
		Yes	181 (55·0%)	168 (53·0%)
		No	148 (42·4%)	149 (44·2%)
		Missing	21 (6·0%)	20 (5·9%)
	Diabetes			
		Yes	281 (85·4%)	268 (84·5%)
		No	48 (13·8%)	49 (14·5%)
		Missing	20 (5·7%)	20 (5·9%)
Surgery type				
	Vascular		314 (90·0%)	305 (90·5%)
	Colorectal		9 (2·6%)	14 (4·2%)
	Plastics		2 (0·6%)	1 (0·3%)
	Other		21 (6·0%)	17 (5·0%)
	Missing		3 (0·9%)	0
Urgency of surgery				
	Elective		141 (40·4%)	143 (42·4%)
	Emergency		205 (58·7%)	194 (57·6%)
	Missing		3 (0·9%)	0
Contamination level of surgery				
	Clean		94 (26·9%)	77 (22·8%)
	Clean contaminated		36 (10·3%)	30 (8·9%)
	Contaminated		26 (7·4%)	20 (5·9%)
	Dirty		190 (54·4%)	210 (62·3%)
	Missing		3 (0·9%)	0
Wound area, cm^2^			18·30 (8·10–35·00)	18·00 (7·26–33·75)
	<28 cm^2^		243 (69·6%)	231 (68·5%)
	≥28 cm^2^		106 (30·3%)	106 (31·5%)
Wound location				
	Foot		279 (79·9%)	272 (80·7%)
	Leg		40 (11·5%)	29 (8·6%)
	Abdomen		11 (3·2%)	13 (3·9%)
	Other		19 (5·4%)	23 (6·8%)
Number of SWHSIs				
	1		318 (91·1%)	304 (90·2%)
	2		21 (6·0%)	28 (8·3%)
	≥3		7 (2·0%)	5 (1·5%)
	Missing		3 (0·9%)	0
Previous history of SWHSI			57 (17·6%)	51 (16·0%)
SWHSI currently infected (based on clinical opinion)			54 (15·6%)	41 (12·2%)
WHQ score			7·77 (5·28)	8·08 (5·31)
Wound pain score			27·9 (29·8)	26·3 (28·9)

Data are n (%), mean (SD), or median (IQR). NPWT=negative pressure wound therapy. SWHSI=surgical wound healing by secondary intention. WHQ=Bluebelle Wound Healing Questionnaire.

* Participants were asked if they consume alcohol (yes or no) and then subsequently asked their average number of units per week.

Most participants had a single SWHSI (n=622, 90·7%), with most wounds arising after vascular surgery (n=619, 90·2%) and located on the foot or leg (n=620, 90·4%).

Over the 12-month follow-up period, wound healing occurred in 202 (57·9%) participants allocated to NPWT, and 196 (58·2%) participants allocated to usual care. 159 (23·2%) participants were censored (83 [23·8%] in the NPWT group and 76 [22·6%] in the usual care group) due to death, amputation, withdrawal, and loss to follow-up (appendix p 47). The median time to healing was 187 days (95% CI 169–226) in the NPWT group and 195 days (158–213) in the usual care group (difference of 8 days).

**Figure 2:**
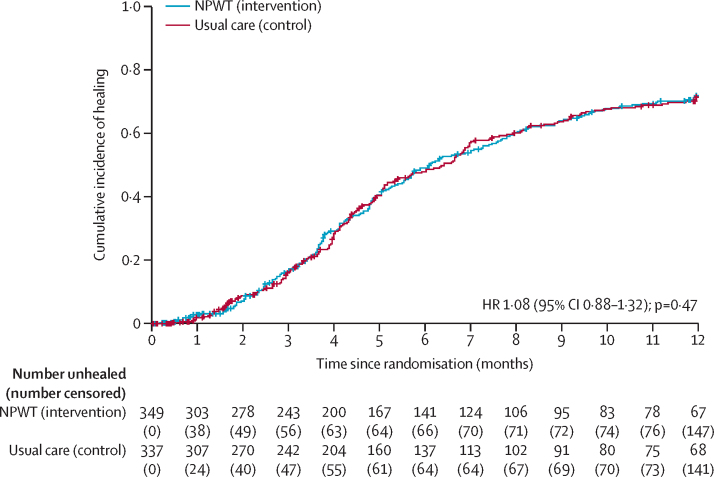
Kaplan–Meier curve of time from randomisation to healing in SWHSI patients receiving NPWT or usual care treatments HR=hazard ratio. NPWT=negative pressure wound therapy. SWHSI=surgical wound healing by secondary intention.

The HR for time to wound healing was 1·08 (95% CI 0·88–1·32; p=0·47; table 2; figure 2), which narrowly includes our target effect size. Results were consistent for sensitivity and subgroup analyses (appendix pp 47–49), and a similar result was observed for the secondary outcome of masked outcome assessment of healing (HR 1·13 [95% CI 0·87–1·47]).

**Table 2:** Primary and secondary outcomes for NPWT and usual care groups for the intention-to-treat population

	**NPWT group**	**Usual care group**	Treatment difference (95% CI)*	**p value**
Primary analysis population	n=349; median time to healing 187 days (95% CI 169 to 226)	n=337; median time to healing 195 days (95% CI 158 to 213)	HR 1·08 (0·88 to 1·32)	0·47
Time to healing as assessed by masked outcome assessment†	n=349; 157 days (95% CI 140 to 188)	n=337; 158 days (95% CI 134 to 203)	HR 1·13 (0·87 to 1·47)	0·36
Hospital admission‡	n=320; 63 (19·7%)	n=320; 58 (18·1%)	OR 1·13 (0·76 to 1·69)	0·54
Reoperation‡	n=320; 78 (24·4%)	n=320; 69 (21·6%)	OR 1·20 (0·82 to 1·74)	0·35
Amputation‡	n=320; 35 (10·9%)	n=320; 36 (11·2%)	OR 0·98 (0·60 to 1·62)	0·95
Wound infection‡	n=320; 102 (31·9%)	n=320; 100 (31·2%)	OR 1·05 (0·75 to 1·48)	0·77
Antibiotic use (for SWHSI)‡	n=320; 211 (65·9%)	n=320; 210 (65·6%)	OR 1·01 (0·70 to 1·45)	0·96
Death‡	n=349; 40 (11·5%)	n=337; 43 (12·8%)	OR 0·89 (0·56 to 1·41)	0·61
WHQ at 3 months§	n=195; mean 7·01 (5·08)	n=190; mean 7·15 (5·72)	Mean adjusted difference 0·29 (−1·01 to 1·58)	0·66
WHQ at 6 months§	n=132; mean 4·71 (4·33)	n=118; mean 4·94 (5·67)	Mean adjusted difference 0·29 (−1·19 to 1·77)	0·70
WHQ at 12 months§	n=86; mean 5·75 (5·76)	n=74; mean 5·52 (5·60)	Mean adjusted difference 1·09 (−0·65 to 2·83)	0·22
Wound pain at 3 months§	n=197; mean 18·9 (24·7)	n=202; mean 17·4 (23·1)	Mean adjusted difference −0·58 (−6·45 to 5·30)	0·85
Wound pain at 6 months§	n=139; mean 17·6 (27·3)	n=124; mean 15·3 (24·3)	Mean adjusted difference −0·28 (−7·11 to 6·54)	0·94
Wound pain at 12 months§	n=90; mean 15·9 (24·4)	n=83; mean 11·5 (18·3)	Mean adjusted difference 1·03 (−7·02 to 9·08)	0·80

HR=hazard ratio. NPWT=negative pressure wound therapy. OR=odds ratio. SWHSI=surgical wound healing by secondary intention. WHQ=Bluebelle Wound Healing Questionnaire.

* Adjusted for wound size, duration of wound, and wound location as fixed effects and centre as a random effect.

† Cox's proportional hazards regression. Data are 25th percentiles as fewer than half of the participants had healing confirmed in this analysis set so a median could not be reported.

‡ Logistic regression using events over 12 months of follow-up.

§ Linear regression.

The primary analysis found a small non-statistically significant reduction (8%) in the time to healing in the NPWT group compared with the usual care group over the 12-month follow-up period. This finding is, however uncertain and NPWT might increase the time to healing by as much as 12% or decrease it by as much as 32%.

A subsequent hospital admission was reported in 63 (19·7%) participants in the NPWT group and 58 (18·1%) participants in the usual care group (odds ratio [OR] 1·13 [95% CI 0·76–1·69]). Return to theatre occurred in 78 (24·4%) participants in the NPWT group and 69 (21·6%) participants in the usual care group (OR 1·20 [95% CI 0·82–1·74]) and amputation occurred in 35 (10·9%) participants in the NPWT group and 36 (11·2%) participants in the usual care group (0·98 [95% CI 0·60–1·62]). Wound infection after randomisation occurred in 102 (31·9%) participants in the NPWT group and in 100 (31·2%) participants in the usual care group (OR 1·05 [95% CI 0·75–1·48]). Antibiotic use for the SWHSI was observed in 211 (65·9%) participants in the NPWT group and 210 (65·6%) participants in the usual care group (OR 1·01 [95% CI 0·70–1·45]). Overall, 40 (11·5%) participants in the NPWT group and 43 (12·8%) participants in the usual care group died (OR 0·89 [95% CI 0·56–1·41]).

There was no evidence of a difference in the patient-reported outcomes (WHQ scores and wound pain) at any timepoint.

Adverse event rates were similar between the two groups (150 [43·0%] of 349 in the NPWT group *vs* 139 [41·2%] of 337 in the usual care group; risk difference –1·7% [95% CI –9·1 to 5·7]; table 3). Wound infection was the most common event type for both groups. 14 serious adverse events were reported (nine participants in the NPWT group and five participants in the usual care group), with major wound infection being the most common event type for both groups. Most events were deemed to be unrelated or unlikely to be related to study treatment (n=10, 71·4%). Further details are provided in the appendix (pp 49–51).

**Table 3:** Adverse and serious adverse events

		**NPWT group (n=349)**	**Usual care group (n=337)**	**Risk difference (95% CI)**
Number of participants with at least one adverse event		150 (43·0%)	139 (41·2%)	−1·7% (−9·1 to 5·7)
Number of patients with adverse events of special interest		48 (13·8%)	47 (13·9%)	0·2% (−5·0 to 5·4)
	Amputation (major)*	33 (9·5%)	38 (11·3%)	..
	Amputation (minor)*	7 (2·0%)	3 (0·9%)	..
	Revascularisation, angioplasty	7 (2·0%)	5 (1·5%)	..
	Revascularisation, angioplasty (unsuccessful)	0	1 (0·3%)	..
	Revascularisation, surgical (common femoral artery endarterectomy)	1 (0·3%)	0	..
Number of participants with at least one serious adverse event		9 (2·6%)	4 (1·2%)	−1·3 (−3·4 to 0·6)
Serious adverse event category				
	Death†	1 (11%)	0	..
	Medication overdose	0	1 (20%)	..
	Revascularisation, angioplasty	1 (11%)	1 (20%)	..
	Wound bleeding	1 (11%)	0	..
	Wound infection (major, debridement)	4 (44%)	0	..
	Wound infection (major, endarterectomy)	0	1 (20%)	..
	Wound infection (major, revascularisation)	0	2 (40%)	..
	Wound infection (moderate)	1 (11%)	0	..
	Wound infection (severe)	1 (11%)	0	..
Total deaths, including those recorded in other questionnaires		40 (11·5%)	43 (12·8%)	1·3% (−3·6 to 6·2)

NPWT=negative pressure wound therapy. SWHSI=surgical wound healing by secondary intention.

* Amputations might not correspond exactly to those included in the secondary outcome as these are amputations at any location regardless of the SWHSI location and at any point during the study (rather than within 12 months of randomisation).

† Deaths may not always have been recorded on serious adverse event forms but we have included all recorded deaths in the study in the table (including those reported in other case report forms).

The within-trial economic analysis estimated an unadjusted mean per-participant QALY and mean per-participant cost of 0·581 (95% CI 0·549 to 0·612) and £5782·13 (95% CI £4151·32 to £7412·95) for the NPWT group and 0·562 (0·528 to 0·595) and £5801·99 (£3897·19 to £7706·78) for the usual care group, respectively. Adjusted mean incremental QALYs and incremental costs suggest that NPWT increased QALYs by 0·007 (95% CI –0·024 to 0·038) and increased costs by £251·44 (95% CI –£2192·63 to £2695·52) over the 12-month study period, although these results were not statistically significant.

The incremental net monetary benefit was estimated to be –£93·22 and –£18·65 at £20 000 and £30 000 cost-effectiveness thresholds, respectively. The probability of NPWT being cost-effective is 47·2% at a £20 000 threshold, and 49·7% at a £30 000 threshold. The cost-effectiveness plane and cost-effectiveness acceptability curve (appendix p 65) graphically present the results of the base-case analysis. Findings from the sensitivity analyses are available in the appendix (pp 65–70).

## Discussion

This open-label, multicentre, pragmatic, parallel-group, randomised controlled trial, primarily involving patients with a lower limb SWHSI, including those with complications of diabetes, found a small non-statistically significant reduction (8%) in the time to healing in the NPWT group compared with the usual care group over the 12-month follow-up period. Results were consistent for sensitivity and subgroup analyses.

There were also no statistically significant differences between groups in the odds of relevant clinical secondary outcomes (readmission, reoperation, infections and antibiotic use, amputation, or death), patient-reported outcomes (WHQ and pain scores), or masked outcome assessment using wound photographs.

The within-trial cost–utility analysis found that, after adjustment for baseline characteristics, baseline EQ-5D index scores and baseline costs, NPWT increased quality of life in patients, and increased costs. However, these results were not statistically significant. The probability of NPWT being cost-effective is less than 50% below the cost-effectiveness thresholds recommended by the UK National Institute for Health and Care Excellence. Sensitivity analysis reduced the probability of cost-effectiveness when participants who died during the study were excluded from the analysis, and when treatment cross-over effects were considered. The complete case analysis suggests that NPWT might be cost-effective, but due to the extent of missing data (75·7%), these findings are likely to be biased.

Despite manufacturers' claims that NPWT promotes wound healing,^8^ this large study has not supported this statement. This finding raises the question of whether NPWT should be used in practice, particularly in the context of healing lower limb SWHSI, including lower limb wounds arising because of complications of diabetes.

A previous Cochrane systematic review^7^ identified only two studies of NPWT as a treatment for patients with SWHSI.^9^, ^11^ Both studies suggested that NPWT reduced the time to wound healing, and there were no differences in infection, amputation, or death rates.^9^, ^11^ Both studies were small and at risk of bias due to limited reporting of healing events. A separate meta-analysis of 48 low-quality studies at high risk of bias suggested a benefit in healing for SWHSI treated with NPWT (OR 1·56 [95% CI 1·15–2·13]; p=0·008), and no difference in infection, amputation, pain, or quality-of-life outcomes between the groups.^28^ The study populations and outcomes in both reviews^7^, ^28^ are not directly comparable with our research and so comparisons cannot be drawn. For example, both reviews include patient populations that are underrepresented in our study, such as those with pilonidal sinus and perivascular groin wounds,^7^ and wounds that were non-surgical in origin (eg, venous leg ulcers or pressure ulcers).^28^ Furthermore, the included studies in both reviews did not adequately assess time to wound healing as an outcome; for example, not including the number of healing events^7^ or using a collective wound closure outcome, which included surgical wound closure.^28^

Considering the limitations in the quality of previous evidence, the SWHSI-2 trial provides the first robust evaluation of the effectiveness of NPWT as a treatment, in terms of time to healing, for patients with an SWHSI. The trial was done in multiple centres, with a large sample size recruited from a diverse range of UK sites. All participants recruited were included in the primary outcome analyses.

Notably, there is an overrepresentation of patients with lower limb wounds, including those with complications of diabetes in the study sample. The study planned to include cross-surgical specialty recruitment; however, equipoise posed a substantial challenge to the recruitment of patients with SWHSI not located on the lower limb. Despite our best efforts, these recruitment difficulties could not be overcome. However, this reflects the pragmatism of the trial as it reflects the populations likely to receive NPWT in practice. Consideration should also be given to the way in which NPWT use differs between surgical specialities and caution in interpretation of the results might be required when applying these findings to SWHSI not located on the lower limb.

This study assessed use of NPWT in relation to time to healing. This population was susceptible to some missing data in the form of censoring due to death and amputation; however, every effort was made to minimise missing data by using a range of methods to collect date of healing before censoring where possible. NPWT can, however, also be used for wound or exudate management. The use of NPWT for these reasons was not assessed in this study, and so conclusions regarding NPWT effectiveness for these purposes are not covered by this study.

Masked outcome assessment was used to minimise subjective assessment of the primary outcome. This secondary analysis did, however, censor wounds deemed unhealed by masked outcome assessors, which has resulted in overestimation of the time to healing. This result should therefore be interpreted with caution.

Patient-reported secondary outcome measures were collected via postal questionnaire, with response rates for these being lower than anticipated. Modifications to the data collection tools and methods had a positive, but limited, impact on response rates (unpublished).

Study recruitment was ongoing at the time of the COVID-19 pandemic restrictions in the UK, resulting in the funder, sponsor, and trial management group mandating a temporary pause to study activity (between March and July, 2020) and site capacity impacts when recruitment and follow-up activities recommenced. We, however, sought to minimise the impacts of this temporary pause to study activity by using agile trial management to support recruiting sites and mitigate impacts where possible.

Finally, given that the SWHSI-2 study was done within the UK, the economic analysis conducted is specific to the UK and so the cost-effectiveness findings might not be generalisable to other countries.

This study has clear implications for commissioners of services and clinicians; NPWT did not confer an advantage in terms of time to healing or adverse events over standard care, particularly for patients with lower limb SWHSI, including those with complications of diabetes. This finding suggests that NPWT should not be considered as a first-line treatment for such patients in relation to wound healing. This finding highlights the challenges that remain in wound care and the clear need to identify new candidate interventions for the healing of SWHSI.

The scarcity of previous randomised controlled trial evidence to support NPWT effectiveness has resulted in an ineffective and costly intervention being used in routine care for a considerable period. The current governance systems allowing this intervention to be used without comparative effectiveness data might not be optimal. The implications for how and when new wound dressings are introduced in practice should therefore be considered by policy makers.

In conclusion, we found no evidence that NPWT reduced the time to wound healing compared with standard dressings in a population of patients with predominantly lower limb wounds, including those with complications of diabetes. Therefore, these findings do not support the use of NPWT to augment SWHSI healing in such patients.

### SWHSI-2 Trial Investigators

### Site investigators

### Contributors

### Data sharing

Anonymised datasets generated and analysed during the current study will be stored in a publicly available open research repository. Data will be made available via this repository after completion of analysis and subsequent publication. Sharing of this anonymised data is covered by original participant consent for the SWHSI-2 trial, which permits sharing of data to support future research via sharing anonymously.

## Declaration of interests

Aside from research funding received for other projects outside of this work, CA reports financial support for attendance to present the study at the Vascular Society of Great Britain and Ireland Annual Scientific Meetings and LM reports participation as an independent member of Data Monitoring and Ethics committees for unrelated studies (REACH-ASD and ACORN II). All other authors declare no competing interests.
