# Supplementary material for: Negative pressure wound therapy versus usual care in patients with surgical wound healing by secondary intention in the UK (SWHSI-2): an open-label, multicentre, parallel-group, randomised controlled trial
Source: Lancet. 2025 May 10;405(10490):1689–99. doi: 10.1016/S0140-6736(25)00143-6 (PMC12062190; doi:10.1016/S0140-6736(25)00143-6)

THE LANCET

Supplementary appendix

This appendix formed part of the original submission and has been peer reviewed. We post it as supplied by the authors.

Supplement to: Arundel C, Mandefield L, Fairhurst C, et al. Negative pressure wound therapy versus usual care in patients with surgical wound healing by secondary intention in the UK (SWHSI-2): an open-label, multicentre, parallel-group, randomised controlled trial. *Lancet* 2025; published online April 15. https://doi.org/10.1016/S0140-6736(25)00143-6.

Appendix - Contents

Study Protocol	Pages 1 to 44
Participating Centres	Page 45
Additional Analyses – Clinical	Page 46 to 52
Additional Analyses – Health Economics	Page 53 to 70

Study Protocol

A pragmatic, multicentre, randomised controlled trial to assess the clinical and cost effectiveness of negative pressure wound therapy versus usual care for surgical wounds healing by secondary intention (SWHSI-2)

Sponsor Reference:

R2319 REC Reference:

IRAS Reference:
258802

Date and Version number: Version 1.6 01.07.2022

Chief Investigator: Professor Ian Chetter

University of Hull, Hull York Medical School and Hull University Teaching Hospitals NHS Trust

Ian.chetter@hey.nhs.uk

Sponsor: Hull University Teaching Hospitals NHS Trust
Office 14, 2nd Floor Daisy Building, Castle Hill Hospital, Cottingham, East Yorkshire, HU16 5JQ

Funder: NIHR Health Technology Assessment Programme (17/42/94)

Trials Unit: York Trials Unit, Department of Health Sciences, University of York, York YO10 5DD

Funding Acknowledgement: This project was funded by the NIHR Health Technology Assessment Programme (17/42/94).

Department of Health and Social Care disclaimer: The views expressed are those of the authors and not necessarily those of the NHS, the NIHR or the Department of Health and Social Care

Collaborators

The following authors / collaborators have assisted with the writing of the protocol:

Professor Ian Chetter	Chief Investigator
Catherine Arundel	Grant Co-applicant/Trial Manager
Karen Armstrong-Lamb	Grant Coapplicant
Jane Blazeby	Grant Coapplicant
Belen Corbacho Martin	Grant Coapplicant/Health Economist
Stephen Dixon	Grant Coapplicant/Patient and Public Representative
Jo Dumville	Grant Coapplicant
Caroline Fairhurst	Grant Coapplicant/Statistician
Eileen Henderson	Grant Coapplicant
Catherine Hewitt	Grant Coapplicant
Matthew Lee	Grant Coapplicant
Rhiannon Macefield	Grant Coapplicant
Angela Oswald	Grant Coapplicant
Thomas Pinkney	Grant Coapplicant
Pedro Saramago-Goncalves	Grant Coapplicant/Health Economist
Nikki Stubbs	Grant Coapplicant
David Torgerson	Grant Coapplicant

Signature Page

Chief Investigator Name: ____________________

Chief Investigator signature: ____________________

Date: ____________________

Sponsor Representative Name: ____________________

Sponsor Representative signature: ____________________

Date: ____________________

Note: All site Principal Investigators and delegated staff must sign their relevant Training Log and be clearly identified on the site Delegation of Authority and Signature log before commencing work on the SWHSI-2 trial.

Amendment History

Amendment No.	Protocol Version No.	Date issued	Author(s) of changes	Details of Changes made
Pre Approval	1.1	21.03.19	Catherine Arundel	- Change to Inclusion criteria relating to participant age. Reduced from 18 years to 16 years at request of the approving Research Ethics Committee
Substantial Amendment 1	1.2	10.09.19	Catherine Arundel	- Clarification on derivation of sample size<br>- Clarification to exclusion criteria<br>- Addition of details of engagement with sites<br>- Addition of wound photograph at Baseline<br>- Removal of CDC assessment at 3 months, in selected sites
Substantial Amendment 2	1.2	Not applicable	Not applicable	- No change to protocol.
Substantial Amendment 3	1.3	28.01.20	Catherine Arundel	- Clarification to exclusion criteria<br>- Addition of two methodological studies within a trial (SWATs)

				- Reduction of post wound healing photographs to one image at initial assessment<br>- Inclusion of patient taken post wound healing photograph where required<br>- Removal of right to erasure in accordance with GDPR for research
Substantial Amendment 4	1.4	20.10.20	Andrew Mott	- Addition of a participant newsletter<br>- Addition of pens with postal CRFs<br>- Changes to participant photography procedure<br>- Addition of telephone follow-up<br>- Addition of PPI group invite
Non Substantial Amendment 16	1.5	15.07.2021	Catherine Arundel	- Change to allow consent to be obtained by any suitably qualified and experience member of the research team as agreed with local R&D and Sponsor.<br>-

Contents

1. Abbreviations 8
2. Background and Rationale 10
   2.1 Background 10
   2.2 Rationale and Justification 10
3. Aims and Objectives 12
   3.1 Aim 12
   3.2 Objectives 12
4. Trial Design 13
   4.1 Design 13
   4.2 Setting 15
   4.3 Outcomes 15
      4.3.1 Primary Outcome 15
      4.3.2 Secondary Outcome 15
      4.4 Studies Within a Trial (SWATs) 16
5.0 Study Population 19
   5.1 Inclusion Criteria 19
   5.2 Exclusion Criteria 19
6.0 Trial Processes 21
   6.1 Participant Identification and Randomisation 21
   6.2 Participant Follow Up 22
      6.2.1 Weekly Telephone Follow Up 25
      6.2.2 Post Healing Follow Up 25
      6.2.3 Postal Questionnaire 26
   6.3 Participant Withdrawal 26
   6.4 Source Data 27
7.0 Study Treatments 28
   7.1 Intervention: Negative Pressure Wound Therapy 28
   7.2 Control: Usual Care (no NPWT) 28
8.0 Adverse Event Management 29
   8.1 Adverse Events (AE) 29

8.2 Serious Adverse Events (SAE) 29
8.3 Reporting Procedures for Adverse and Serious Adverse Events 30
9. Statistical and Health Economics Considerations 31
9.1 Sample Size 31
9.2 Internal Pilot Phase 31
9.3 Statistical Analysis 32
9.4 Health Economic Analysis 33
9.5 SWAT Analysis 34
9.5.1 Recruitment SWAT 34
9.5.2 Retention SWAT 34
10.0 Ethical Arrangements 35
10.2 Risks and Benefits 35
10.3 Informing Participants of Potential Risks and Benefits 36
10.4 End of Trial 36
10.5 Retention of Trial Documentation 36
10.6 Compliance with Medicines Devices Directive 36
11. Trial Finance and Insurance 37
11.1 Trial Funding 37
11.2 Trial Insurance 37
12. Project Management 38
12.1 Trial Sponsor 38
12.2 Trial Management 38
12.3 Trial Management Group 38
12.4 Trial Steering and Data Monitoring Committees 38
12.5 Patient and Public Involvement (PPI) 39
13. Dissemination and projected outputs 41
14. References 42

1. Abbreviations

**AE**	Adverse Event
**CACE**	Complier Average Causal Effect
**CI**	Chief Investigator
**CONSORT**	Consolidated Standards of Reporting Trials
**CRF**	Case Report Form
**DMC**	Data Monitoring Committee
**EQ-5D-5L**	EuroQol 5 dimension 5 level questionnaire
**GCP**	Good Clinical Practice
**GP**	General Practitioner
**HTA**	Health Technology Assessment Programme
**ISF**	Investigator Site File
**KCI**	Kinetic Concepts
**LPLV**	Last Patient Last Visit
**MRC**	Medical Research Council
**MUST**	Malnutrition Universal Screening Tool
**NHS**	National Health Service
**NICE**	National Institute for Health and Care Excellence
**NIHR**	National Institute for Health Research
**NPWT**	Negative Pressure Wound Therapy
**PAG**	Patient Advisory Group
**PI**	Principal Investigator
**PPI**	Patient and Public Involvement
**PIS**	Patient Information Sheet
**RCT**	Randomised Controlled Trial
**REC**	Research Ethics Committee
**SAE**	Serious Adverse Event

**SSI**	Surgical Site Infection
**SWHSI**	Surgical Wounds Healing by Secondary Intention
**TMF**	Trial Master File
**TMG**	Trial Management Group
**TSC**	Trial Steering Committee
**WHQ**	Wound Healing Questionnaire
**YTU**	York Trials Unit

2. Background and Rationale

2.1 Background

More than 10 million surgical operations are performed in the NHS every year (1). Wounds are usually closed by apposing the wound edges - "healing by primary intention". When closure is not possible, or when primarily closed wounds break down (dehisce), they are usually left open to heal from the bottom up, through formation of granulation tissue - "healing by secondary intention".

Surgical wounds healing by secondary intention (SWHSI) are a common, complex problem. A recent survey estimated the UK prevalence of SWHSI to be 4.1 per 10,000 population, and identified colorectal, plastics and vascular as the surgical specialities most commonly associated with SWHSI (2).

Treatment of these wounds presents a significant financial burden to the NHS, with costs of SWHSI estimated to be £1,060 per patient, before inclusion of treatment costs. The healing pathway of SWHSI patients is often prolonged and complex with a variety of treatment options, ranging from basic, relatively inexpensive wound dressings (around £441 per month) to complex, expensive treatments such as Negative Pressure Wound Therapy (NPWT, around £1,323 per month (3)).

These wounds pose a unique management challenge; they can remain open for many months, may require a multitude of treatments, are highly susceptible to infection, and may require prolonged hospitalisation and/or further operations (4). The healing pathway of patients with an open wound is therefore often prolonged and complex, which has a substantial impact on patient quality of life (5, 6). In a recent cohort study (400 SWHSI patients), the median time to healing was 86 days (95% CI 75 to 130) and in specific wound sites (e.g. foot/leg) the duration was almost double that of wounds elsewhere on the body (7). Infection (32.1%), hospital re-admission (24.7%) and further surgical procedures (16.8%) were all common (7).

SWHSI patients often require frequent dressing changes, and the use of complex expensive treatments like NPWT has increased; from 6% to 29% of SWHSI in a one-year period (8).
NPWT was developed in the 1990s as a treatment for full thickness wounds. The device applies a controlled negative pressure (vacuum) to a wound via a specialist dressing, removing wound fluid into a canister (9). KCI, the manufacturer who pioneered the use of NPWT in their V.A.C® device, claim that the mechanical forces generated by the negative pressure create a wound environment that is conducive to healing by removing infective materials and exudate, reducing oedema and promoting perfusion and granulation (http://www.kci1.com/KCI1/sciencebehindwoundtherapy). NPWT is not anticipated to be used to the point of healing, rather its use is promoted as part of the treatment pathway to reduce the time taken for healing to be achieved.

2.2 Rationale and Justification

Ongoing and previous research has investigated the use of NPWT, in complex chronic and closed wounds, primarily for prevention of surgical site infections (10-15). Despite the increasing number of trials assessing NPWT, there has been limited research evaluating NPWT as a treatment for SWHSI. Recent systematic reviews

have identified only three randomised controlled trials (RCTs) assessing NPWT as a treatment for SWHSI; however, all were small, and with caution advised with regards the interpretation of findings (9, 18).

Two small RCTs comparing NPWT with standard care for SWHSI (combined total n = 69), were identified in a recent Cochrane systematic review (9). The data were poorly analysed and reported, and thus presented uncertain evidence for the effectiveness of NPWT compared with standard care in terms of time to healing. Other data (e.g. adverse events) were also limited (9). One of the two included trials compared NPWT with an alginate dressing in participants with a groin SWHSI following arterial surgery (16). Median time to healing was shorter in the NPWT group (n=10, 57 days, range 25 to 115) than in the alginate dressing group (n=10, 104 days, range 57 to 175). Analysis of time to event data was, however, not undertaken appropriately so was not considered robust. The second trial compared NPWT with silicone dressing in participants who had undergone pilonidal sinus excision (17). Median time to healing was shorter in the NPWT group (n=24, 84 days, range 34 to 349) than in the dressing group (n=25, 93 days range 43 to 264). The review authors urge caution in interpretation of these findings, given it was unclear how the analysis was undertaken. The review concluded that there was no rigorous RCT evidence for the clinical effectiveness of NPWT in the treatment of SWHSI and that potential benefits and harms remain uncertain.

A second Cochrane review of NPWT for treating diabetic foot wounds identified a further RCT of NPWT versus dressings in participants who had a SWHSI following diabetic foot amputation (18). Median time to healing was significantly shorter in the NPWT group (n=43, 56 days, range 26 to 92) than in the dressing group (n=33, 77 days, range 40 to 112) (19). Caution in interpretation was again recommended given this RCT included patients with adequate foot perfusion (therefore not necessarily representative of patients with diabetic foot wounds), was commercially funded, and was deemed to be at risk of performance bias (18).

A further study taking place in Germany (20) to assess the effectiveness of NPWT for abdominal SWHSI has been identified however results are not yet publically available.

Given this evidence, NPWT as a treatment for SWHSI has been introduced into clinical practice without high quality evidence supporting its clinical or cost effectiveness. Given the increasing use of this device in routine care, a full and sufficiently powered randomised controlled trial is essential to evaluate the effectiveness of this treatment for SWHSI. The SWHSI-2 trial has therefore been designed as a pragmatic, multi-centre randomised controlled trial to evaluate the clinical and cost effectiveness of NPWT vs usual care for the treatment of SWHSI.

3. <u>Aims and Objectives</u>

3.1 Aim

To assess the clinical and cost-effectiveness of Negative Pressure Wound Therapy (NPWT) as compared to usual care (no NPWT) in treating surgical wounds healing by secondary intention (SWHSI).

3.2 Objectives

i. To include a six-month internal pilot phase to obtain robust estimates of recruitment rates and confirm trial feasibility.

ii. To undertake a parallel group randomised controlled trial to test the hypothesis that NPWT is superior to usual care (no NPWT) in treating SWHSI based on time to healing in days from randomisation.

iii. To conduct a detailed economic evaluation to compare the cost-effectiveness of NPWT to usual care (no NPWT) to determine the most efficient provision for future care and resources.

4. Trial Design

4.1 Design

SWHSI-2 is a pragmatic, multi-centre, cross surgical specialty, two-arm, parallel group, pragmatic, randomised controlled, superiority trial. The study includes an internal pilot phase to assess recruitment assumptions and optimise trial processes.

The study has a 24-month recruitment period, including an internal pilot followed by the main recruitment period. Following randomisation, participants will receive weekly clinical telephone follow up and outcome assessments will be conducted at 3, 6, and 12 months post randomisation. A flow diagram demonstrating the patient pathway through the study is provided in Figure 1.

Neither patients nor health care practitioners will be blinded to treatment allocation as the treatments cannot be adequately concealed. The primary outcome (time to healing – defined using the commonly used and clinically certified criteria '*complete epithelial cover in the absence of a scab (eschar)*') will however be verified by independent, blinded observers using standardised photographs.

The trial will be pragmatic: the inclusion criteria will be relevant to a broad range of patients and wounds; NPWT device, wound dressing and co-intervention choices (including but not limited to dressings and wound packing, antimicrobial gels and emollients, and debridement) will be left to the discretion of the clinical care team; and all types of SWHSI will be eligible for inclusion, provided that the patient is otherwise appropriate.

Figure 1 – Study Flow Diagram

Patients with a surgical wound healing by secondary intention - *Identified in participating centres in secondary care and community NHS Trusts and primary care centres*

Inclusion Criteria

- Aged 16 years or over
- An acute SWHSI, of less than six weeks in duration, arising from any surgical specialty, and occurring on any part of the body, deemed appropriate to receive either NPWT or wound dressing treatment
- Wound is considered ready for NPWT treatment (i.e. contains at least 80% viable tissue or has only a very thin layer of slough requiring no further debridement)
- Patient is not deemed to be malnourished (as per NICE guidelines CG32 or assessed as at high risk using the MUST Tool)
- Patient is willing and able to give informed consent and provide follow up data.

Exclusion Criteria

Patient: life expectancy <6 months; active systemic infection; inadequate haemostasis/risk of bleeding; requires emergency airway aspiration, pleural mediastinal or chest tube drainage or surgical suction; currently participating in another wound research study where the primary outcome time point has not been reached.

Wound: chronic wounds non-surgical in origin; wound is currently or has previously been treated with NPWT; planned delayed primary closure; contraindication to NPWT (e.g. unclear undermining in the wound, presence of necrotic or malignant tissue or eschar, involves exposed blood vessels or nerves, organs or anastomotic sites, located where vacuum seal cannot be obtained, presence of non-enteric of unexplored fistula)

Baseline

Demographics, comorbidity, smoking status, and surgical procedure details. Wound Photograph.
Wound Pain, Quality of Life (EQ-5D-5L), Bluebelle Wound Healing Questionnaire, Resource Use

Randomise

696 patients

Negative Pressure Wound Therapy

348 patients

Using any CE marked device, in use within the NHS. The clinical care team will determine the duration of device use and treatment regimen.

Usual Care (No NPWT)

348 patients

Treatment with any dressing type. The clinical care team will determine the dressing type and frequency of dressing change.

3 months Follow Up

Wound pain
Quality of Life (EQ-5D-5L)
Bluebelle WHQ
Resource use data

6 month Follow Up

Quality of Life (EQ-5D-5L)
Resource use data

12 months Follow Up

Quality of Life (EQ-5D-5L)
Resource use data

Weekly Telephone Assessment (to point of healing)
Wound healing
Clinical events (e.g. infection, return to hospital, further procedures, death)
Treatment status
Adverse events

Following wound healing (on 3 consecutive weeks from healing)
Clinical verification of healing
Photographs of wound for blinded outcome assessment (Visit 1 only)
Bluebelle Wound Healing questionnaire (Visit 1 only)
CDC reference assessment (Visit 1 only)

4.2 Setting

The study will enlist at least 20 sites (NHS hospitals, community NHS Trusts and primary care centres) to recruit one to two patients per site per month, over the two-year recruitment period. This rate is based on experience within our recent, feasibility RCT (RP-PG-0609-10171) (21).

We will incentivise participating sites by offering collaborative authorship, for named co-investigators, where recruitment, on average, exceeds one patient per month at that site (22).

We will work closely with participating sites from the outset to engage relevant surgical specialties to promote the study and encourage recruitment activity.

4.3 Outcomes

4.3.1 Primary Outcome

The primary outcome is time to healing in days from randomisation (defined using the commonly used and clinically certified criteria '*complete epithelial cover in the absence of a scab (eschar)*').

Confirmation of wound healing by a health care professional will initially be participant-reported through weekly telephone contact with the research nurse. Participants will be asked to report if their clinician or nurse has indicated that their wound is healed, and will be asked to confirm that this meets the healing definition (i.e. there is full coverage and no scab present). In the event the wound healing has not been confirmed by a healthcare professional, the research nurse will contact the clinical care team to obtain this confirmation.

Once wound healing has been confirmed by a healthcare professional and treatment has ceased, participants will undergo clinical assessments on three subsequent consecutive weeks to confirm continued wound healing. A standardised photograph will be taken of the wound at the first healing assessment to enable blinded outcome verification. The first healing visit will be completed face to face wherever possible. We will also ask participants to take and return a photograph of the wound themselves. Study specific instructions will be provided to the participant, and they will be encouraged to ask a friend or relative to assist with this should the wound location mean it is difficult for them to take the photograph independently.

Blinded outcome verification is crucial for studies with subjective outcomes such as healing and infection (23). Therefore, time to healing within the SWHSI-2 trial will be verified using photographs by clinically experienced, independent, blinded observers in order to prevent reporting bias. A robust photography protocol, and associated training, will be established to ensure standardisation of imaging throughout the trial.

4.3.2 Secondary Outcome

- Clinical events including antibiotic treatment, hospital admission or discharge, treatment status (including reasons for dressing or treatment failure or change), re-operation (including skin grafting and closure surgery*), amputation and death.

  * The decision for closure surgery will be made blinded to treatment allocation as far as possible.

- Wound infection: Assessed using the Bluebelle Wound Healing Questionnaire (WHQ) (24).The questionnaire includes items to assess signs, symptoms and

wound care interventions indicative of surgical site infection (SSI) and can be completed by patient self-report or by healthcare professionals. The tool may be used to assess wounds in hospital or after the patient has been discharged. The WHQ will be completed by the participant themselves at baseline, 3month follow up assessment, and will also be completed by the patient at the initial healing visit.

The WHQ has been validated for primary closed wounds in a cohort of 800 patients receiving abdominal surgery (25). Inclusion of the WHQ in this study will provide valuable validation data for its use in patients with SWHSI. A reference SSI assessment (using the Centres for Disease Control and Prevention (CDC) classification for SSI) will be collected as part of the WHQ validation. The research nurse will complete the reference assessment after the participant has completed the WHQ at the initial assessment visit following wound healing.

- Pain: A visual analogue scale will be used to assess wound pain (with anchors 0 'no pain' and 10 'worst imaginable pain'). The pain scale will be completed by the participant themselves at baseline and 3month follow up assessment.

- Quality of Life: Information on quality of life will be collected at baseline and 3, 6 and 12 months, post randomisation using EQ-5D-5L (26) generic instrument consistent with NICE recommendations. The EQ-5D-5L measures health related quality of life in terms of five dimensions: mobility, ability to undertake usual activities, pain and discomfort, anxiety, and depression.

- Resource use: Wound-related NHS consultations, support (e.g. occupational therapy,) and out of pocket costs will be collected using a patient reported questionnaire at baseline, 3, 6 and 12 months. Details of wound dressing changes (frequency and type) will be collected at weekly follow up.

In addition, epidemiological data including patient demographics (including date of birth, gender, ethnicity), comorbidities, smoking status, surgical procedure details (e.g. urgency, contamination level (27), type), and a wound photograph will be collected at baseline.

4.4 Studies Within a Trial (SWATs)

We will undertake two studies within a trial (SWATs) to assess the effectiveness for methods to improve recruitment and retention. Strategies have been registered, or will be registered with the Medical Research Council SWAT repository prior to activity commencing.

4.4.1 Recruitment SWAT

We will evaluate the effects of presentation of the study design to participants on recruitment rate. Participants will be randomised (on a site basis) to receive an infographic (visual document explaining the study) plus the standard patient information sheet (PIS) or just the PIS.

A recent review showed that tailoring or shortening the patient information sheet given to participants makes little or no difference to recruitment (28). None of the studies included in the review, however, tested the use of information graphics ("infographics") to enhance recruitment. The evidence around the effectiveness of infographics in a health context is limited but persuasive. Infographics have been shown to improve patient knowledge; both in relation to personally relevant information such as discharge instructions, and statistical information such as the association of age with cancer risk (29) (30). While a further study with patients, students and doctors found that infographics did not increase knowledge when compared to plain language summaries the infographics did improve reader experience and user-friendliness (31)

These findings suggest that there may be the potential for infographics to increase potential participant experience and understanding of health research leading to increased recruitment.

As is usual with embedded trials, the sample size is constrained by the number of patients approached about the study hence a formal power calculation to determine sample size has not been conducted.

This will be a cluster trial; randomisation will be carried out at the site level to reduce cross contamination. The allocation ratio will be 1 to 1. Generation of the allocation sequence will be undertaken independently by a researcher not involved with the recruitment of participants. Randomisation will utilise minimisation of the following factors: i) whether the site is recruiting cross specialty or in a single specialty; and ii) expected number of eligible participants as reported on the site feasibility assessment, cut at the median.

The primary outcome of this embedded trial will be the recruitment rate, i.e. the proportion of participants in each group who are randomised into the host trial. Secondary outcomes will include the proportion of patients in each group who are screened but do not go on to be randomised, and the cost effectiveness of the intervention.

4.4.2 Retention SWAT

We will evaluate the effect of a thank you card sent in advance of follow up questionnaires. Participants will be randomised to receive either the thank you card or no thank you card. Cards will be sent at Month 4 and Month 9 following recruitment.

Many recruitment and retention strategies routinely include some element of thanks within them. Although we often do this, there is little to no evidence to suggest this might work. Recent evidence suggests that those saying thank you often undervalue its effect (32). There is therefore a need to test the impact of thanks in the context of trial recruitment and retention.

As is usual with embedded trials, the sample size is constrained by the number of patients actively participating within the host trial, hence a formal power calculation to determine sample size has not been conducted.

All participants recruited into the SWHSI-2 trial, who remain as fully participating (i.e. have not fully withdrawn, withdrawn from postal follow up or have died) and who have yet to reach the 4 month time point will be eligible for the SWAT. There are no additional inclusion or exclusion criteria.
Generation of the allocation sequence will be undertaken independently by a researcher not involved with the follow up of participants. Participants will be allocated 1:1 using block randomisation, stratified by the host trial's treatment arm, using randomly varying block sizes to avoid imbalance between the SWAT intervention arms.

The primary outcome of this embedded trial will be questionnaire response rate, i.e. the proportion of participants who return their questionnaire in each group. Secondary outcomes will include whether a reminder notice is required, completeness of response and cost of the intervention per participant retained.

5.0 Study Population

We will include all adult patients with a SWHSI who fulfil all of the Inclusion Criteria detailed, and none of the Exclusion Criteria below.

5.1 Inclusion Criteria

1) Aged 16 years or over.

2) Has an acute SWHSI *(i.e. a wound left open as planned following surgery or a wound initially closed using sutures, clips, or other closure methods and dehisced along the whole or part of its length, and of less than 6 weeks in duration)*, arising from any surgical specialty and occurring on any part of the body, deemed appropriate to receive either NPWT or wound dressing treatment.

3) Has a SWHSI that is considered ready for NPWT treatment (i.e. contains at least 80% viable tissue or has only a very thin layer of slough requiring no further debridement).

4) Patient is not deemed to be malnourished, as per NICE guidelines CG 32 (33) (BMI <18.5 kg/m^2 ; unplanned* weight loss >10% in the last 3-6 months; BMI <20kg/m^2 and unplanned* weight loss >5% in the last 3-6 months) or assessed as at high risk of malnutrition using the Malnutrition Universal Screening Tool (MUST) (34). **Patients with weight loss arising either from underlying comorbidity (e.g. ulcerative colitis) or from the reasons for surgery being completed (e.g. bowel cancer) may be included at the clinician's discretion.*

5) Willing and able to give informed consent and provide follow-up data.

5.2 Exclusion Criteria

1) Life expectancy of less than 6-months e.g. undergoing end stage palliative care.

2) Active systemic infection (including osteomyelitis) at baseline as defined by clinical and/or laboratory assessment. *Note: Patients who have an active infection, but are improving following 1 week's duration of antibiotics may be included at the clinician's discretion.*

3) Inadequate haemostasis or patients who are at risk of bleeding.

4) Chronic wounds non-surgical in origin (e.g. pressure ulcers or foot ulcers*).
*Note diabetic foot ulcers which have been incised and drained, or debrided as an inpatient in theatre may be included given this constitutes a surgical wound

5) Current open wound has previously been, or is currently being, treated with NPWT.

6) Planned delayed primary closure of the wound.

7) Contraindication to NPWT including:

a. Presence of unclear undermining in the wound cavity (i.e. the deepest point of the wound cannot be measured)
b. Presence of necrotic tissue, malignant tissue or eschar
c. Wounds involving exposed blood vessels and/or organs, anastomotic sites and/or nerves (including the “open abdomen” where the abdominal fascia is open)
d. Wounds situated where, in the opinion of the treating clinician, a vacuum seal cannot be obtained.
e. Presence of a non-enteric or unexplored fistula
f. People requiring emergency airway aspiration, pleural mediastinal or chest tube drainage or surgical suction.

8) Currently participating in another wound research study, where the primary outcome has not yet been reached.

6.0 Trial Processes

6.1 Participant Identification and Randomisation

Screening to identify eligible patients for the trial will initially occur in the surgical departments of participating NHS hospitals. Patients may also be recruited from community NHS trusts or primary care settings.

Patients with a potential planned SWHSI (pre-operatively) or a SWHSI occurring at any point following surgery will be screened for potential eligibility by their clinical care team or GP. The research team will work closely with the treating clinicians at each participating site to optimise the local screening and recruitment processes.

Potential participants will be approached with further details of the trial, including an information sheet. This may be completed in person by the surgeon, regular nurse or research nurse during a ward round, routine care or home visit.

It will be clearly stated that the participant is free to withdraw from the study at any time for any reason without prejudice to future care, and with no obligation to give the reason for withdrawal. Should new information arise during the study which may affect a participant's willingness to take part, this will be reviewed for addition to the patient information sheet and a revised consent form will be completed as necessary.

The participant will be allowed as much time as they wish to consider the information, and given the opportunity to question the Principal Investigator, the research team, their GP or other independent parties to decide whether they will participate in the study.

Potential participants will then be shown the consent form and will be given the opportunity to ask questions about the study.

Informed consent will be obtained by a suitably qualified and experienced member of the research team, as agreed by the participating site R&D group and/or Sponsor if required, and who has been authorised to do so by the Chief or Principal Investigator, as detailed on the study Delegation of Authority and Signature Log for the study site. The participant must personally sign and date the latest approved version of the informed consent form before any study specific, baseline procedures are performed.

The original signed form will be retained at the study site within the Investigator Site File (ISF); while copies will be given to the participant, retained in the participant's medical notes, and provided to the study coordinating centre.

Once informed consent has been obtained, baseline data will be collected. This includes:

- Surgical Site Infection (Bluebelle Wound Health Questionnaire (WHQ))
- EQ-5D-5L
- Pain (using a Visual Analogue Scale)
- Epidemiological data (e.g. demographics, comorbidities, smoking status, surgical procedure details)
- Wound location and area
- Photograph of the patients wound.

A delegated member of the research team, will then contact York Trials Unit (YTU), by telephone or via the internet, to access a secure randomisation service. The randomisation service will record information and check eligibility to avoid inappropriate entry of patients into the trial. Independent random allocation 1:1 (NPWT:Usual Care), stratified by wound location (foot (i.e. hind, mid or fore foot areas) and ankle), leg (i.e. upper leg, lower leg and knee), abdomen, other), wound

area (<28cm^2, ≥28cm^2), and centre will then be completed. Variable block sizes within strata will be used. Treatment will be allocated on an individual named patient basis and the patient started on treatment as soon as possible after randomisation.

Where patients are screened pre-operatively, consent will be obtained pre-operatively and randomisation completed either in theatre or post operatively. Baseline data will be collected post operatively.

6.2 Participant Follow Up

Participants will receive weekly clinical follow-ups for the purposes of the study, and will be asked to complete participant self-reported questionnaires at 3, 6, and 12 months post randomisation. Details of assessments are summarised below and in the study procedure summary in Figure 2.

Two weeks before their 6 month follow-up participants will be sent a study newsletter, this will provide an update on the study progress and answer questions the participant may have about the study at that time. This has been shown to increase retention.(35)

Figure 2 – Study Procedure Summary

	**Enrolment**	**Allocation**					
**TIMEPOINT**	**Pre-randomisation/ baseline**	**Randomisation**	**Weekly Telephone Contact to point of Healing**	**Postal Questionnaire 3 month post-randomisation**	**Postal Questionnaire 6 month post-randomisation**	**Postal Questionnaire 12 month post-randomisation**	**Post Healing Assessment visits (x3)**
**ENROLMENT**							
Eligibility screen	X						
Informed consent	X						
Baseline questionnaire	X						
Allocation		X					
**ASSESSMENTS**							
Wound Healing			X				X
Wound Photographs	X						X
Dressing Changes			X				
Clinical Events			X				
Adverse events			X				

Bluebelle WHQ	X			X			X
CDC assessment							X
EQ-5D-5L	X			X	X	X	
Pain Visual analogue scale	X			X			
Resource use	X			X	X	X	

6.2.1 Weekly Telephone Follow Up

Randomised participants will be contacted weekly by telephone, on a pre-agreed day and time, to assess:

- Wound healing (defined using the commonly used and clinically certified criteria '*complete epithelial cover in the absence of a scab (eschar)*')
- Clinical events
- Treatment Status
- Adverse events.

Information collected will be recorded in a case report form (CRF). Where a participant cannot be contacted, further contact attempts will be made on the following two consecutive days.

6.2.2 Post Healing Follow Up

When the participant reports that their clinician or nurse has indicated that their wound is healed, and it is confirmed that this meets the healing definition (i.e. there is full coverage and no scab present), the research nurse will undertake an assessment with the participant, within 48 hours of the report of healing being made, to confirm that the wound has healed. In the event the wound healing has not been confirmed by a healthcare professional, the research nurse will first contact the clinical care team to obtain this confirmation.

Clinical assessments will then be completed with the participant on three consecutive weeks (commencing with the initial healing visit). To enable a standardised photograph to be taken at the initial healing visit for blinded outcome verification, a range of follow up delivery options will be used:

1) Wherever possible the first healing visit is completed face to face, by the research nurse, with the participant at home, or in a clinical care setting if preferred.
2) If face to face contact cannot be facilitated (e.g. due to patient availability, local or national government guidelines), NHS approved video call technology should be used to undertake assessment, with a screen shot taken of the wound.
3) If video call technology cannot be used then a telephone call will be undertaken to assess healing, with the participant asked to take and return a photograph of their wound.

Subsequent healing visits (weeks 2 and 3) may then be completed by telephone, if preferred, to confirm continued wound healing.

At the initial healing assessment, we will also ask participants to take and return a photograph of the wound themselves. Study specific instructions will be provided to the participant, and they will be encouraged to ask a friend or relative to assist with this should the wound location mean it is difficult for them to take the photograph independently.

The WHQ will be completed and a CDC assessment will also be performed by the research nurse (after the participant has completed the WHQ) at the initial assessment visit following wound healing.

6.2.3 Postal Questionnaire

Participants will also be followed up by postal questionnaire, sent by York Trials Unit, at 3, 6 and 12 months post randomisation to collect:

- EQ-5D-5L (26)
- Pain (using a Visual Analogue Scale) (Month 3 only)
- Surgical site infection (Bluebelle WHQ (24)) (Month 3 only)
- Resource use.

Participants will be provided with a freepost envelope to facilitate return of the completed questionnaire to York Trials Unit for processing. Participants will also be provided with a pen to aid completion of the questionnaire, this has been shown to increase the number of questionnaires returned.(36)

Should it not be possible to post a questionnaire to the participant the participant will be contacted by telephone by York Trials Unit to complete the questionnaire.

Where no response is provided to a questionnaire, a reminder letter will be sent to the participant after two weeks to encourage completion and return of the questionnaire; this has been shown to increase the likelihood of response (37).

Where no response is provided to the questionnaire or reminder after a further two weeks, the participant will be contacted by telephone by York Trials Unit to complete the questionnaire.

Use of incentives have been found to be effective in facilitating the return of postal questionnaires (37). We will therefore include a monetary incentive of £5 with both the 6 month and 12 month questionnaires. Participants will be pre-notified of this unconditional token in the letter that accompanies their initial questionnaire (at 3 months).

6.3 Participant Withdrawal

Each participant has the right to withdraw from the study at any time without prejudice. In addition, the investigator may advise that a participant be discontinued from the study at any time if the investigator considers it necessary for any reason, however the decision on full withdrawal will remain with the participant at all times.
The reason for withdrawal will be recorded in a CRF. If the participant is withdrawn due to an adverse event, the investigator will complete follow-up visits or telephone calls until the adverse event has resolved or stabilised.

Participants who request to fully withdraw during a study assessment will be asked if they are willing to complete the questionnaires prior to withdrawal. Where a participant fully withdraws outside of a scheduled study visit, completion of further follow up questionnaires will not be requested.

If the participant withdraws consent, they will be asked to confirm the elements they wish to withdraw from i.e. all data collection, postal data collection or telephone data collection. Where a participant requests withdrawal from all or telephone data collection, the participant will be asked if the research team can try to obtain outcome data through contact with healthcare professionals. Where full withdrawal is requested, patients will be asked if they are happy for their personal details to continue to be stored.

Where participants lose capacity to consent during their time in the study, they will be withdrawn from further follow up however data collected until this point will be retained for use. No further data would be collected or any other research procedures conducted in relation to the participant.

6.4 Source Data

Source documents are original documents, data, and records from which participants' CRF data are obtained. These include, but are not limited to, hospital records (from which medical history and previous and concurrent medication may be summarised into the CRF), clinical and office charts, diaries, correspondence, completed scales and quality of life questionnaires.

CRF entries will be considered source data if the CRF is the site of the original recording (e.g., there is no other written or electronic record of data). In this study the CRF will be used as the source document as outlined in the Source Data Verification form.

All documents will be stored safely in confidential conditions. Any paper forms containing participant identifiable information (e.g. patient details form and consent form) will be held in a location separate to the questionnaire data. Identifiable information will be stored securely in a locked filing cabinet, in an office only accessible via registered swipe card access held by the York Trials Unit research team (As per YTU Standard Operating Procedure YT03).

Personal data held electronically, will be stored on the study specific participant management system which will record identifiable information and participant activity to enable study coordination. This will be accessible via individual password. Permissions for access of this information will also be detailed within the study delegation log. The server on which the management system will be housed is secure and is subject to rigorous testing and continued backup.
A photograph collected to record participant wound healing at the initial post wound healing assessment visits will be anonymised prior to electronic transfer by sites to the University of York, where the image will be stored in an encrypted and password protected drive. All data will be stored in accordance with data protection principles.

On all study-specific documents, other than the signed consent, the participant will be referred to by the study participant number/code, not by name.

7.0 Study Treatments

7.1 Intervention: Negative Pressure Wound Therapy

Negative Pressure Wound Therapy (NPWT) consists of a computer-controlled vacuum pump into which disposable components fit. A disposable, plastic canister slots into the pump to collect wound exudate. The canister is attached to pressure resistant tubing that connects to the wound dressing creating an airtight seal, and is removed and replaced either when it becomes full, or at least once a week.

The wound is filled with a suitable dressing, for example: black, polyurethane foam dressings with reticulated (open) pores to help evenly distribute negative pressure across the wound bed; white, polyvinyl alcohol foam with high tensile strength, pre-moistened with sterile water; and antimicrobial gauze (impregnated with Polyhexamethylene biguanide). The choice of dressing can depend on factors such as the size, shape and location of the wound, the quantity and viscosity of the exudate, availability, patient and professional preferences and experience. To ensure the pragmatic nature of the trial, dressing selection will be left to the discretion of the clinical care team.

Negative pressure or suction is achieved in the wound cavity by using either a drain (flat or channel) or a port connected to the tubing. The wound is sealed with a transparent adhesive polyurethane film. Occasionally a liner is placed in the wound bed prior to the application of foam or gauze to protect vulnerable structures such as blood vessels or organs and prevent dressing adherence.

The device is generally used as part of the SWHSI treatment pathway rather than to the point of healing and is administered by both nurses and clinicians.

The use of any CE marked NPWT device, providing pressure of 60-150mmHg, in use within the NHS will be permitted in this trial, given that the principles of any device are similar and there is no evidence to suggest clinical or cost effectiveness differences between devices. The device will be used in accordance with manufacturer guidance, and the clinical care team, in conjunction with local treatment guidelines, will determine the duration of device use, and whether this includes continuous or intermittent pressure cycles. Treatment regimen and details will be recorded during weekly telephone follow up.

7.2 Control: Usual Care (no NPWT)

Usual care will be that used locally, without NPWT. This is most likely to be other sorts of wound dressings.

Given that there is no evidence to suggest any one dressing is more clinically and cost effective than another (38), use of any dressing type will be permitted. The clinical care team will determine the dressing type (primary and secondary) and frequency of dressing change and details will be recorded will be recorded during weekly telephone follow up.

8.0 Adverse Event Management

8.1 Adverse Events (AE)

For the purposes of the SWHSI-2 trial, adverse events (AE) are defined as any untoward medical occurrence (i.e. any unfavourable and unintended sign, symptom or disease), experienced by a clinical trial participant and which is temporally associated with study treatment (interventions or control) and is related to the wound or to the study intervention or control treatments.

Adverse events, which might be expected with these wounds include minor wound infection, cellulitis, oedema, maceration and retention of product in the wound e.g. wound filler embedding in granulated tissue.

8.2 Serious Adverse Events (SAE)

To ensure no confusion or misunderstanding of the difference between the terms "serious" and "severe", which are not synonymous, the following note of clarification is provided:

The term "severe" is often used to describe the intensity (severity) of a specific event (as in mild, moderate, or severe myocardial infarction); the event itself, however, may be of relatively minor medical significance (such as severe headache). This is not the same as "serious," which is based on patient/event outcome or action criteria usually associated with events that pose a threat to a participant's life or functioning. Seriousness (not severity) serves as a guide for defining reporting obligations.

Serious adverse events are defined as any untoward medical occurrence that:

1) Results in death

2) Is life threatening

NOTE: The term "life-threatening" in the definition of "serious" refers to an event in which the participant was at risk of death at the time of the event; it does not refer to an event that hypothetically might have caused death if it were more severe.

3) Requires unplanned inpatient hospitalisation or prolongation of existing inpatients' hospitalisation

4) Results in persistent or significant disability or incapacity

5) Is a congenital anomaly or birth defect

6) Any other important medical condition that, although not included in the above, may require medical or surgical intervention to prevent one of the outcomes listed.

For the purposes of the SWHSI-2 trial, hospitalisation for the treatment of major wound infection, osteomyelitis, wound bleeding, fistulation, for removal of embedded wound filler and for limb amputation, will **not** be considered a SAE but will be reported using the SWHSI-2 Adverse Event Form.

8.3 Reporting Procedures for Adverse and Serious Adverse Events

Adverse events (AE) should be entered onto the Adverse Event reporting form and reported to York Trials Unit within 5 days of discovery or notification of the event.

Serious adverse events (SAE) should be entered onto the Serious Adverse Event reporting form and reported to York Trials Unit within 24 hours of discovery or notification of the event. Once received, causality and expectedness of serious adverse events will be confirmed by the Chief Investigator or another clinical member of the Trial Management Group (if the CI is unavailable).

SAEs that are deemed to be unexpected and related to the trial will be notified to the Research Ethics Committee (REC) and Sponsor within 15 days. All such events will be reported to the Trial Steering Committee and Data Monitoring Committee at their next meetings.

All events will be followed up until the event resolves or a decision is made for no further follow up. Participants experiencing SAEs which are deemed to be related to the trial treatments (intervention or control) and which remain ongoing at the time of participant trial exit will be followed up for one further month beyond trial exit.

Where repeated adverse events (serious or non-serious) of a similar type are observed, these will be discussed with the Data Monitoring Committee (DMC) and will be onward reported to the REC and Sponsor should concerns be raised in relation to the type of event and/or frequency observed.

9. Statistical and Health Economics Considerations

9.1 Sample Size

A conservative estimate of a 25% decrease in median time to healing, assuming a median time to healing of 86 days in the usual care group, between the two treatment groups will be sought. This equates to a 21-day reduction in time to healing to 65 days in the NPWT group.

To detect a 25% reduction in median time to healing (from 86 days with usual care to 65 days with NPWT), with 90% power, and allowing for 20% attrition, 696 participants are required to be recruited and randomised (348 NPWT; 348 Usual Care).

The 25% reduction in time to healing used here, has been selected on the following basis:

- Cost Effectiveness: Models generated using observational data obtained in our previous cohort study suggest a 57.4% difference in time to healing would be required to demonstrate cost effectiveness of NPWT (3). This should however be interpreted with caution given this is derived from observational data.

- Current Literature: The average median time to healing in the control group of previous observational and RCT studies is 86 days, with an average decrease in time to healing of 25% (16, 17, 19, 39).

- Significance to Patients: Patients are frequently disappointed by the slow healing process of a SWHSI and complete wound healing is therefore a major focus for patients (6). Patient representatives have confirmed that the proposed reduction in time of 21 days with NPWT is likely to be significant for patients.

The proposed attrition rate used here, is derived from rates observed in previous studies: RP-PG-0609-10171 cohort study (n=66/393, 16.8%) (7); Armstrong et al (n=38/162, 23%) (19); Blume et al (n=103/341, 30%) (40).

Recruiting 696 patients, and assuming an attrition rate of 20%, we would also have over 80% power to detect an absolute difference of 10% in the percentage of participants experiencing wound healing if the healing rate in the control group was between 20% and 80%.

9.2 Internal Pilot Phase

The first six months of recruitment will constitute an internal pilot phase and will be evaluated on the following predefined criteria to ascertain our ability to recruit and randomise.

1) To set up at least 10 sites
2) To randomise 100 patients (on average, one to two patients per site per month)
3) 80% of patients to receive intervention within 48 hours of randomisation
4) Feasibility of follow up (>80% response rate to 3-month questionnaire)

Reasons for treatment discontinuation will also be monitored.

Recruitment assumptions and intervention rate will be assessed initially at 3 months, and again at 6 months. Feasibility of follow up and preparing of study sites to open for recruitment will be completed at 6 months to allow sufficient data to be collected.
Assumptions will be assessed against pre-defined ‘traffic light’ stop go criteria:
Green – Recruitment rate and intervention rate >80%; Non response to month 3 questionnaire ≤15%
Amber – Recruitment and intervention rate 60-80%; Non response to month 3 questionnaire 15 – 20%
Red – Recruitment and intervention rate <60%; Non response to month 3 questionnaire > 20%.

9.3 Statistical Analysis

Full details for the analysis will be provided in a statistical analysis plan, which will be prepared by the trial statistician and reviewed by the Trial Management Group, and Trial Steering and Data Monitoring Committees prior to the completion of data collection. Analyses are described in brief here.

This trial will be reported according to the CONSORT guidelines for clinical trials (41). A CONSORT diagram will be produced to show the flow of participants through the trial, providing reasons for non-participation and withdrawal where available (41).

Analysis will be undertaken using Stata version 15 or later (College Station, TX: StataCorp LLC).

Baseline data will be summarised by randomised group, as randomised and as included in the primary analysis (42), using descriptive statistics for continuous variables (n, mean, standard deviation, median, minimum, and maximum) and count and percentage for categorical variables. No formal statistical comparison of baseline data will be made between the groups.

All outcomes will be summarised descriptively by randomised group. Outcome analyses will be conducted following the principles of intention-to-treat with participant’s outcomes analysed according to their original, randomised group irrespective of deviations based on non-compliance.

Significance tests will be two-sided at the 5% significance level. Parameter estimates will be presented with associated 95% confidence intervals (CI) and p-values as appropriate. For all outcomes, the level of missing data and the missing data mechanism will be assessed, and if required appropriate imputation techniques will be considered.

Our primary outcome is time to healing of the reference wound derived as the difference in days between randomisation and the first date of complete healing. Healing rates will be presented overall and by trial arm. Kaplan–Meier survival curves will be produced for the two groups and the median time to healing with a 95% CI presented. A proportional hazards Cox regression model will be used to compare the healing times between the two groups, adjusting for wound size at

baseline, duration of wound in days (time between wound start date and randomisation), and wound location as fixed effects, and centre as a shared frailty effect. To assess the impact of compliance on the primary outcome we will consider a Complier Average Causal Effect (CACE) analysis which will produce an unbiased estimate of the treatment effect in the presence of non-compliance (defined as participants in the NPWT group who do not receive NPWT). Participants in the standard care group who receive NPWT will be considered as a cross-over.

Secondary outcomes, including adverse events, will be analysed where appropriate using regression techniques appropriate for the type of data.

9.4 Health Economic Analysis

The economic analysis will aim to evaluate the lifetime cost-effectiveness of NPWT, compared to all relevant comparators, in the treatment of SWHSI. The perspective of the cost-effectiveness analysis will be of the UK NHS and the Personal Social Services. The primary economic outcome will be the incremental cost-effectiveness ratio for NPWT vs alternatives, expressed as cost per Quality Adjusted Life Years (QALYs) gained.

To best assist decision-making, we aim to build a *de-novo* decision analytic model to establish which of the relevant treatment(s) are most cost-effective, with current information. Evidence taken from the SWHSI-2 RCT will be key to inform parameters of the economic model. The wider existing evidence base, identified through literature reviews, will also be used. As the decision analytic model will require accommodating multiple different and relevant information sources, when required and suitable, evidence synthesis techniques will be used to pool data to best inform specific decision model input parameters.

Regression approaches will be used to derive costs and health benefits (health utility measured using EQ-5D-5L), allowing for correlation between these as well as adjusting for key covariates. Alternative scenarios regarding the extrapolation of the primary outcome over the lifetime of the model, and the evidence informing it, will be explored. Uncertainty in the evidence base used to populate the decision analytic model will be characterised using appropriate distributions and any uncertainty in the adoption decision will be demonstrated using probabilistic sensitivity analysis. The value of further data collection will be established using value of information analysis.

9.5 SWAT Analysis

9.5.1 Recruitment SWAT

Primary analysis: The primary analysis, the difference in recruitment rate between those receiving the infographic in addition to the PIS and those not receiving the infographic, will be analysed via logistic regression adjusting for the factors used in the minimisation, with site as a random effect.

For the secondary analyses, the difference in proportion of those responding to a recruitment invitation who received the infographic in addition to the PIS but who do not go on to be randomised, and those not receiving the infographic but who do not go on to be randomised will also be analysed via logistic regression adjusting for the factors used in the minimisation, with site as a random effect.

The difference in cost per recruited participant between those offered the infographic and those not offered the infographic will be calculated. In addition to the direct costs of the infographic, it may also be necessary to include the cost of staff time spent administering the recruitment packs.

9.5.2 Retention SWAT

The primary analysis, the difference in retention rate at 6 months between those receiving the thank you card and those not receiving a thank you card, will be analysed via logistic regression adjusting for main trial allocation, and site as a random effect.

For the secondary analyses, the difference in completeness of response between those receiving the thank you card and those receiving no thank you card will be analysed via linear regression adjusting for main trial allocation, and site as a random effect. The difference in the proportion of participants requiring a reminder letter mailing between those receiving a thank you card and those receiving no thank you card will be analysed via logistic regression adjusting for main trial allocation, and site as a random effect.

The difference in cost per retained participant between those sent a thank you card and those not sent the thank you card will be calculated. In addition to the direct costs of the thank you card and postage, it may also be necessary to include the cost of staff time spent administering the mail out (for example filling and labelling envelopes).

The secondary outcomes at 12 months will be analysed as described above for the 6-month outcomes.

10.0 Ethical Arrangements

10.1 Ethical Approval

The SWHSI-2 trial will be conducted in accordance with the Clinical Trials Regulations (2004/1031) and will be subject to approval from the Research Ethics Committee and the Health Research Authority prior to study activity commencing. The study will be conducted in accordance with the Research Governance Framework and Medical Research Council (MRC) Good Clinical Practice (GCP) Guidance (43, 44).

Before being enrolled in the SWHSI-2 trial, participants must consent to participate after the nature, scope, and possible consequences of participating in the clinical study have been explained in a form understandable to them. The Investigator will not undertake any measures specifically required only for the clinical study until valid consent has been obtained.

A Patient Information Sheet (PIS) that includes information about the study and a consent form will be given to the participant. These documents will contain all the elements required by the ICH E6 Guideline for GCP and any additional elements required by local regulations. The PIS will give a balanced account of the possible benefits and known risks of the interventions. It will state explicitly that quality of care will not be compromised if the participant decides to a) not enter the trial or b) withdraw their consent. We will make it clear that there is no obligation to participate.
Patients will be given the opportunity to ask questions and the nature and objectives of the study will be explained. At the time of consent, written informed consent must be confirmed by the personally dated signature of the participant and the delegated member of research team conducting the informed consent discussions.

The original signed consent form will be retained in the study files. Other copies of the consent form are required:

- One copy of the informed consent form will be sent securely to YTU (by secure fax or encrypted email) and filed in the Trial Master File.
- One copy of the informed consent form will be kept in the patient’s clinical notes where applicable. If a patient does not have clinical notes at the trial site, the informed consent document will be filed in a separate folder.
- One copy will be given to the patient.

Consent is an ongoing process and will be reassessed at each study visit.

10.2 Risks and Benefits

Side effects for NPWT or wound dressings are uncommon, and both treatments are routinely used in the NHS for patients with SWHSI. Risks to participants because of any of the treatments are not increased through trial participation.

There are however some associated risks with NPWT. This treatment uses a portable machine which may present a trip hazard for patients and their families. The nature of NPWT also means that dressing or foam used for this treatment may adhere to the wound surface, particularly as granulated tissue forms. Where patients are recruited with an abdominal wound, there is also a potential risk of fistulation. Patients will be advised of these risks in the information sheet.

10.3 Informing Participants of Potential Risks and Benefits

Informed consent will be obtained by a delegated member of research team using a detailed patient information sheet developed with the help of service users, which will explain the risks and benefits clearly. In the unlikely event that new information arises during the trial that may affect participants' willingness to take part, this will be reviewed by the Trial Steering Committee for addition to the patient information sheet. A revised consent form will also be completed if necessary.

10.4 End of Trial

The end of the SWHSI-2 trial will be the Last Patient Last Visit (LPLV), defined as:

- Completion of 12 month follow up assessment in the study
- Withdrawal from follow up due to any reason

10.5 Retention of Trial Documentation

In line with the principles of Good Clinical Practice/UK Clinical Trials Regulations, essential Trial documentation will be kept with the Trial Master File and Investigator Site Files. This documentation will be retained for a minimum of 5 years after the conclusion of the trial to comply with standards of Good Clinical Practice, and Sponsor requirements.

Case Report Forms will be used to record all the information required from the protocol and will be stored for a minimum of 5 years after the conclusion of the trial as paper records (stored in a secure storage facility either on or off-site) and a minimum of 5 years in electronic format (on a password protected server) in accordance with guidelines on Good Research Practice (44).

10.6 Compliance with Medicines Devices Directive

The techniques under investigation are in routine use within the NHS and are internationally accepted surgical procedures using CE-marked implants and medical devices. We do not therefore require prior authorisation by the UK Competent Authority, the MHRA, under the Medical Devices Regulations (2002).

11. Trial Finance and Insurance

11.1 Trial Funding

The SWHSI-2 trial is funded by the NIHR Health Technology Assessment (HTA) Programme (HTA Reference: 17/42/94).

The Schedule of Events and Statement of Activity approved by the Health Regulatory Authority details all related costings for the SWHSI-2 trial.

All interventions are standard treatment options currently available in the NHS. We anticipate therefore that there will be no excess treatment costs for these interventions.

11.2 Trial Insurance

The Clinical Negligence Scheme for Trusts is able to provide insurance to cover for liabilities and prospective liabilities arising from negligent harm. Clinical negligence indemnification will rest with the participating NHS Trust or Trusts under standard NHS arrangements.

12. Project Management

12.1 Trial Sponsor

The trial will be sponsored by Hull University Teaching Hospitals NHS Trust.

12.2 Trial Management

York Trials Unit (YTU) at the University of York will manage the study. YTU will also provide quality assurance for trial processes through centralised monitoring of key trial procedures (e.g. consent and eligibility) and documentation completion, in addition to routine checks with sites with regards documentation held in Investigator Site Files.

Each site will have a site Principal Investigator (PI) who will be responsible locally for the study. All trial staff will be trained in the trial procedures by YTU during site set up, thereby meeting the Sponsors (and NIHR) standards. Where required by the NHS Trust site, trial staff will have current GCP certification.

To ensure continued engagement with, and to provide support to, participating research sites, bi-monthly meetings will be convened and all active research sites invited to participate. Meetings will include sharing best practice in relation to recruitment and retention, problem solving, and sharing any key information relating to the trial or to emerging evidence in relation to open surgical wounds or associated treatments.
A Twitter account (@SWHSI2_Trial) will also be used to provide updates on progress to study sites, and to engage the wider research community with the trial.

The Trial manager, on behalf of the Chief Investigator, will submit and, where necessary, obtain approval from all relevant parties for all substantial amendments to the original approved documents.
Regular progress reports will be submitted as required to the Funding Body.

12.3 Trial Management Group

A Trial Management Group (TMG) will monitor the day-to-day management of the trial including the detailed design, set up, initiation and supervision of the study. This will comprise the Chief Investigator (CI), all co-applicants, trial team at YTU, trial statistician, and trial health economist. A representative of the Sponsor will also be invited to attend. The group will meet bi-monthly (as a minimum) from the start of the study to the end of the pilot phase and quarterly thereafter to manage the detailed design, set up, initiation and supervision of the study.

12.4 Trial Steering and Data Monitoring Committees

Independent oversight of the study will be conducted by the Trial Steering Committee (TSC), who will monitor the progress of the trial and provide independent advice. The TSC will comprise of independent clinicians and health service researchers with appropriate expertise and an independent patient

representative. The TSC meetings will also be attended by the trial statistician and the study Sponsor will be invited to attend.

The study will be regularly reviewed by the Data Monitoring Committee (DMC), comprising of independent clinicians and health service researchers with appropriate expertise. The DMC will monitor the data arising from the study and recommend whether there are any ethical or safety reasons why the trial should not continue.
Both the TSC and DMC will meet at regular intervals to provide project oversight to the trial.

12.5 Patient and Public Involvement (PPI)

Patients and the public have been involved in the development of this study in a number of ways:

- Our Patient Advisory Group (PAG), established for nearly a decade, has contributed to the development of this study through involvement in an earlier NIHR Programme Grant for Applied Research (PGfAR (RP-PG-0609-1017)) which has been used to develop this application.

- Patients have also identified the importance of this research question; qualitative semi-structured interviews with SWHSI patients identified wound healing as the most important outcome measure (6).

- An experienced patient representative has been actively involved in the development of this study, providing input on proposed follow-up methods and outcomes, feedback on application drafts and in writing the plain English summary.

Patient and public involvement (PPI) will continue to be integral throughout the conduct and dissemination of the trial.

We will engage with our Patient Advisory Group (PAG) on a bi-annual basis and will ask them to contribute thoughts on the design, delivery, management and interpretation of this trial. The group will have the opportunity to: review all patient related documentation, specifically to explain the risks and benefits of this research clearly; to review case report forms to ensure all aspects of care important to patients are captured; to review procedures including the consent process; and to make ongoing recommendations as necessary to help improve recruitment and retention.

Independent patient representatives will also participate in the Trial Management Group and Trial Steering and Data Monitoring Committees contributing to trial design, conduct and dissemination. Financial support for PPI attendance at meetings will be provided through reimbursement of time and travel at rates recommended by INVOLVE (45).

At the end of the trial, the management team will develop a patient friendly version of the results summary, based on trial findings. We will ask service users

(as represented by the PAG) to review and revise this so that it is appropriate for a lay audience.
The PAG will assist in developing content for updating the entry on Wikipedia and will help to write the Map of Medicine entry on SWHSI management. In this way, service users will actively participate in dissemination of the conclusions of this research in a manner that is easily accessible by other patients. Representatives from the PAG may also assist with presenting the findings of this research at the Wounds Research Network or Tissue Viability Service meetings, or at local patient focused groups.

We do not anticipate any formal training requirements for our PPI representatives, however they will be directed to, and will have access to, further information regarding involvement in research from INVOLVE (46).

The SWHSI-2 research team are keen to develop the existing PPI group to give patients the opportunity to take part in informing future research studies. All participants who were consented and randomised to participate in the trial (and did not subsequently withdraw from follow-up) will be informed of this opportunity by post at the 12 month follow-up. They will be given the opportunity to be included in this group to inform the dissemination of SWHSI-2 findings and further development of this research area. If interested, participants will be asked to return a completed consent to contact form and the research team will contact them to discuss this group in further detail.

13. Dissemination and projected outputs

Results from this study will be written up and submitted to peer-reviewed journals, irrespective of the magnitude or direction of effect. A publications policy will be generated in advance to detail authorship, acknowledgements and review processes for any publications arising from the SWHSI-2 trial.

The executive summary and copy of the trial report will be sent to the National Institute for Health and Care Excellence (NICE) and other relevant bodies, including Clinical Commissioning Groups, so that study findings can be translated into clinical practice nationally. We will also work with the relevant National Clinical Director in the Department of Health to help ensure the findings of the trial are considered when implementing policy and will work with the Speciality Advisory Committees (SAC) to incorporate the findings into the training curriculum for clinicians who will undertake treatment of SWHSI.

A summary of the study report will be produced and made available to participants, members of our patient advisory group and relevant patient-focused websites. Patient information will also be generated for “Shared Decision Making”, the entry on Wikipedia and the Map of Medicine entry. Service users involved in the SWHSI-2 patient advisory group will be asked to actively participate in dissemination of the conclusions of this study to ensure these are easily accessible to patients.

14. References

1. NHS Confederation. Key statistics on the NHS 2016 [Available from: http://www.nhsconfed.org/resources/key-statistics-on-the-nhs.

2. Chetter IC OA, Fletcher M, Dumville JC and Cullum NA. A survey of patients with surgical wounds healing by secondary intention; an assessment of prevalence, aetiology, duration and management. Journal of Tissue Viability. 2016;26(2):103-7.

3. Saramago-Goncalves PR CK, Welton N, and Soares M. Bayesian Econometric Modelling of Observational Data for Cost-effectiveness Analysis: Establishing the Value of Negative Pressure Wound Therapy in the Healing of Open Surgical Wounds (unpublished). 2018.

4. Mees J. MW, Senninger N, Bruewer M, Palmes D, Mees ST. Treatment options for postoperatively infected abdominal wall wounds healing by secondary intention. Langenbecks Arch Surg. 2012;397(8):1359-66.

5. Sandy-Hodgetts K CK, and Leslie GD. Determining risk factors for surgical wound dehiscence: a literature review. International Wound Journal. 2013;12(3):265-75.

6. McCaughan D SL, Dumville J, Cullum N, and Chetter I. Patients' perceptions and experiences of living with a surgical wound healing by secondary intention: an in-depth qualitative study. International Journal of Nursing Studies. 2018;77:29-38.

7. Chetter IC OA, McGinnis E, Stubbs N, Arundel C, Buckley H, et al. Patients with surgical wounds healing by secondary intention: a prospective, cohort study. International Journal of Nursing Studies. 2018.

8. Chetter IC AC, Bell K, Buckley H, Claxton K, Corbach B, et al. Surgical wounds healing by secondary intention: characterising and quantifying the problem, identifying effective treatments, and assessing the feasibility of conducting a randomised controlled trial of negative pressure wound therapy versus usual care. 2017.

9. Dumville JC OG, Crosbie EJ, Peinemann F and Lui, Z. Negative pressure wound therapy for treating surgical wounds healing by secondary intention. Cochrane Database of Systematic Reviews. 2015;4(6).

10. Team WT. UK WHIST – Wound Healing in Surgery for Trauma - Protocol. 2016 [Available from: https://njl-admin.nihr.ac.uk/document/download/2009661.

11. Gillespie BM. WJ, Ellwood D., Stapleton H., Whitty JA., Thalib L., Cullum N., Mohammed K., Chaboyer W. ADding negative pRESSure to improve healING (the DRESSING trial): a RCT protocol. BMJ Open. 2016;6:e010287.

12. Achten J. PN, Bruce J., Petrou S., Tutton E., Willett K., Lamb SE., Costa ML. Protocol for a randomised controlled trial of standard wound management versus negative pressure wound therapy in the treatment of adult patients with an open fracture of the lower limb: UK Wound management of Open Lower Limb Fractures (UK WOLFF). BMJ Open. 2015;5:e009087.

13. Team WT. WHISH - What is the rate of deep infection after hip fracture surgery in patients with negative pressure wound therapy compared to standard care wound management? [Available from: https://www.octru.ox.ac.uk/trials/trials-open-to-recruitment/whish.

14. Team MT. ISRCTN64926597 - Comparing treatments for diabetic foot ulcers 2017 [Available from: http://www.isrctn.com/ISRCTN64926597?q=&filters=&sort=&offset=5&totalResults=15875&page=1&pageSize=50&searchType=basic-search.

15. Hashmi F. ISRCTN51433523 - Effect of FlowOx™ treatment on healing of lower limb ischaemic ulcers 2017 [Available from: http://www.isrctn.com/ISRCTN51433523.

16. Monsen C W-HC, Wictorsson C, Acosta S. Vacuum-assisted wound closure versus alginate for the treatment of deep perivascular wound infections in the groin after vascular surgery. Journal of Vascular Surgery. 2014;59:145-51.
17. Ulas Biter L BG, Mannaerts GHH, Stok MM, Van Der Ham AC, Grotenhuis BA. The use of negative-pressure wound therapy in pilonidal sinus disease: a randomized controlled trial comparing negative-pressure wound therapy versus standard open wound care after surgical excision. Diseases of the Colon and Rectum. 2014;57(1406-1411).
18. Dumville JC. HR, Cullum N, Game F, Stubbs N, Sweeting M, et al. Negative pressure wound therapy for treating foot wounds in people with diabetes mellitus. Cochrane Database of Systematic Reviews. 2013;10.
19. Armstrong DG aLL. Negative pressure wound therapy after partial diabetic foot amputation: a multicentre, randomised controlled trial. Lancet. 2005;366(9498):1704-10.
20. Seidel D LR, and Neugebauer EA. Treatment of subcutaneous abdominal wound healing impairment after surgery without fascial dehiscence by vacuum assisted closure™ (SAWHI-V.A.C.®-study) versus standard conventional wound therapy: study protocol for a randomized controlled trial. Trials. 2013;14(394).
21. Arundel C FC, Corbacho-Martin B, Buckley H, Clarke E, Cullum N et al. Pilot, feasibility randomized clinical trial of negative pressure wound therapy versus usual care in patients with surgical wounds healing by secondary intention BJS Open. 2018;2(3):99-111.
22. Lee MJ. Recognising contributions to work in research collaboratives: Guidelines for standardising reporting of authorship in collaborative research. International Journal of Surgery. 2017.
23. Hjrobjartsson A. TB, Boutron I., Brorson S. Observer bias in randomised clinical trials with binary outcomes: systematic review of trials with both blinded and non-blinded outcome assessors. BMJ. 2012;344.
24. Macefield RC RB, Milne TK, Nicholson A, Blencowe NS, Calvert M, et al. Development of a single, practical measure of surgical site infection (SSI) for patient report or observer completion. Journal of Infection Prevention. 2017.
25. Group BS. Validation of the Bluebelle Wound Healing Questionnaire for assessment of surgical site infection in closed primary wounds after hospital discharge. British Journal of Surgery. 2018.
26. Brooks R RR, and De Charro F. The measurement and valuation of health status using EQ-5D: A european perspective. Dordrecht, The Netherlands: Kluwer Academic Publishers; 2003.
27. Mangram AJ. HT, Pearson ML, Silver LC, and Jarvis WR. The Hospital Infection Control Practices Advisory Committee. Guideline for prevention of surgical site infection. Infection Control Hospital Epidemiology. 1999;20:247-77.
28. Treweek S, Pitkethly M, Cook J, Fraser C, Mitchell E, Sullivan F, et al. Strategies to improve recruitment to randomised trials. The Cochrane Library. 2018.
29. Hill B, Perri-Moore S, Kuang J, Bray BE, Ngo L, Doig A, et al. Automated pictographic illustration of discharge instructions with Glyph: impact on patient recall and satisfaction. Journal of the American Medical Informatics Association. 2016;23(6):1136-42.
30. McCrorie AD, Chen JJ, Weller R, McGlade KJ, Donnelly C. Trial of infographics in Northern Ireland (TINI): Preliminary evaluation and results of a randomized controlled trial comparing infographics with text. Cogent medicine. 2018;5(1):1483591.
31. Buljan I, Malički M, Wager E, Puljak L, Hren D, Kellie F, et al. No difference in knowledge obtained from infographic or plain language summary of a Cochrane systematic review: three randomized controlled trials. Journal of clinical epidemiology. 2018;97:86-94.
32. N KAaE. Undervaluing gratitude: expressers misunderstand the consequences of showing appreciation. Psychological Science 2018;29(9):1423-35.
33. Excellence NIfHaC. Nutrition support for adults: oral nutrition support, enteral tube feeding and parenteral nutrition. In: Excellence NIfHaC, editor. 2017.

34. Elia M. The MUST report: Nutritional screening of adults a multidisciplinary responsibility. Development and use of the 'Malnutrition Universal Screening Tool' ('MUST') for adults. In: Nutrition BAfPaE, editor. Worcestershire, UK: BAPEN; 2003.
35. Mitchell N, Hewitt CE, Lenaghan E, Platt E, Shepstone L, Torgerson DJ, et al. Prior notification of trial participants by newsletter increased response rates: a randomized controlled trial. J Clin Epidemiol. 2012;65(12):1348-52.
36. James S, Parker A, Cockayne S, Rodgers S, Fairhurst C, Torgerson D, et al. Including a pen and/or cover letter, containing social incentive text, had no effect on questionnaire response rate: a factorial randomised controlled Study within a Trial. F1000Research. 2020;9.
37. Edwards PJ RI, Clarke MJ., Di Guiseppi C.,Wentz R., Kwan I., et al. Methods to increase response to postal and electronic questionnaires. Cochrane Database of Systematic Reviews. 2009(3).
38. Vermeulen H UD, Goossens A, de Vos R and Legemate DA. Systematic review of dressings and topical agents for surgicalwounds healing by secondary intention. British Journal of Surgery. 2005;92:665-72.
39. Danne J. GS, McKenzie D., Danne P. A Retrospective Study of Pilonidal Sinus Healing by Secondary Intention Using Negative Pressure Wound Therapy Versus Alginate or Gauze Dressings. Ostomy Wound Management. 2017;63(3):47-53.
40. Blume PA, Walters J, Payne W, Ayala J, J L. Comparison of negative pressure wound therapy using vacuum-assisted closure with advanced moist wound therapy in the treatment of diabetic foot ulcers: a multicenter randomized controlled trial. Diabetes Care. 2008;31(4):631-6.
41. Moher D. HS, Schulz KF., Montori V., Gotzsche PC., Devereaux PJ., et al. CONSORT 2010 Explanation and Elaboration: updated guidelines for reporting parallel group randomised trials. BMJ. 2010;340.
42. Dumville J, Torgerson, DJ., Hewitt, CE. Reporting attrition in randomised controlled trials. BMJ. 2006;332(969).
43. Department of Health. Research governance framework for health and social care. In: Health Do, editor. UK2005.
44. MRC Ethics Series. Good research practice: principles and guidelines. 2012.
45. INVOLVE. Payment and recognition for public involvement 2018 [Available from: http://www.invo.org.uk/resource-centre/payment-and-recognition-for-public-involvement/.
46. INVOLVE. How to get involved 2018 [Available from: http://www.invo.org.uk/find-out-more/getting-involved/.

Participating Centres

SWHSI-2 Recruiting NHS Trusts
Aneurin Bevan University Health Board (Bwrdd Iechyd Prifysgol Aneurin Bevan)
Bradford Teaching Hospitals NHS Foundation Trust
Royal Cornwall Hospitals NHS Trust
Doncaster and Bassetlaw Teaching Hospitals NHS Foundation Trust
The Dudley Group NHS Foundation Trust
Frimley Health NHS Foundation Trust
Hull University Teaching Hospitals NHS Trust
Imperial College Healthcare NHS Trust
NHS Lanarkshire
The Leeds Teaching Hospitals NHS Trust
Liverpool University Hospitals NHS Foundation Trust
NHS Lothian
Manchester University NHS Foundation Trust
Mid Yorkshire Teaching NHS Trust
The Newcastle upon Tyne Hospitals NHS Foundation Trust
Northumbria Healthcare NHS Foundation Trust
Norfolk and Norwich University Hospitals NHS Foundation Trust
North Bristol NHS Trust
Northern Care Alliance Foundation Trust
The Queen Elizabeth Hospital King's Lynn NHS Foundation Trust
Royal Free London NHS Foundation Trust
The Shrewsbury and Telford Hospital NHS Trust
St George’s University Hospitals NHS Foundation Trust
University Hospitals Birmingham NHS Foundation Trust
University Hospitals of Derby and Burton NHS Foundation Trust
University Hospitals of Leicester NHS Trust
Worcestershire Acute Hospitals NHS Trust

Additional Analyses - Clinical

Additional baseline table

Table 1: Descriptive summary by site

	NPWT (n=349)	Usual care (n=337)
Site ID		
10	77 (22·8%)	78 (22·3%)
11	1 (0·3%)	0 (0·0%)
12	28 (8·3%)	29 (8·3%)
13	13 (3·9%)	12 (3·4%)
14	20 (5·9%)	23 (6·6%)
15	12 (3·6%)	11 (3·2%)
16	18 (5·3%)	19 (5·4%)
17	7 (2·1%)	8 (2·3%)
18	21 (6·2%)	24 (6·9%)
19	5 (1·5%)	5 (1·4%)
20	11 (3·3%)	14 (4·0%)
21	6 (1·8%)	7 (2·0%)
22	5 (1·5%)	7 (2·0%)
23	1 (0·3%)	1 (0·3%)
24	14 (4·2%)	15 (4·3%)
25	15 (4·5%)	14 (4·0%)
26	3 (0·9%)	3 (0·9%)
27	2 (0·6%)	2 (0·6%)
28	8 (2·4%)	7 (2·0%)
29	4 (1·2%)	4 (1·1%)
30	29 (8·6%)	31 (8·9%)
32	7 (2·1%)	6 (1·7%)
33	4 (1·2%)	5 (1·4%)
34	3 (0·9%)	2 (0·6%)
35	6 (1·8%)	4 (1·1%)
36	4 (1·2%)	3 (0·9%)
37	9 (2·7%)	10 (2·9%)
38	4 (1·2%)	5 (1·4%)

Additional Analysis Information

Two additional sensitivity analyses were conducted to include the manual wound size calculation and to remove any covariates that violated the proportional hazards assumption according to the Therneau Grambsch test. [1]

Censoring in the primary analysis

Table 2: Summary of censoring during the study period

	NPWT (n=349)	Usual care (n=337)	Total (N=686)
Censored	83 (24%)	76 (23%)	159 (23%)
Unhealed at 12 months	64 (18%)	65 (19%)	129 (19%)
Healed by 12 months	*202 (58%)*	*196 (58%)*	*398 (58%)*
**Reason for censoring**			
Amputation	34 (41%)	33 (43%)	67 (42%)
Death	24 (29%)	29 (38%)	53 (33%)
Lost to follow up (censored at date of last weekly follow up)	11 (13%)	10 (13%)	21 (13%)
No event data reported (censored at day 1)	14 (17%)	4 (5%)	18 (11%)

Table 3: Full model output from the primary analysis model

	Time to healing (days)		
Coeffcient	*Estimates*	*Conf. Int (95%)*	*P-Value*
Intervention (NPWT)	1·08	0·88 – 1·32	0·472
SWHSI size	0·98	0·98 – 0·99	**<0·001**
SWHSI duration	1·00	0·98 – 1·01	0·781
Wound location: Leg	2·89	1·99 – 4·21	**<0·001**
Wound location: Abdomen	4·42	2·39 – 8·14	**<0·001**
Wound location: Other	2·93	1·93 – 4·44	**<0·001**
$N_{trialsiteid}$	28		
Observations	686		

Sensitivity analysis of the primary outcome

Table 4: Sensitivity analyses on primary outcome

Description of analysis	**n**	Hazard Ratio (95% CI)	p-value
**Pre-planned (specified in Statistical analysis plan)**			
Competing risks model (death and amputation of SWHSI as competing events)	686	0·99 (0·80 to 1·24)	0·93
Wound location corrected (stratification errors)	686	1·10 (0·90 to 1·33)	0·38
Baseline imbalances (including smoking status and alcohol consumption)	686	1·08 (0·88 to 1·33)	0·45

Removal of covariates that violate the proportional hazards assumption	686	1·04 (0·85 to 1·27)	0·69
CACE analysis (using received NPWT as a complier), an alternative to per protocol, detailed in the Statistical Analysis Plan	686	1·08 (0·81 to 1·43)	0·61
**Post-hoc**			
Wound area calculation	686	1·08 (0·88 to 1·32)	0·48
Interval censoring (using mid-point from date healed and last recorded visit)*	686	1·07 (0·87 to 1·31)	0·51

* On the advice of the statistical reviewer

Forest plot by strata

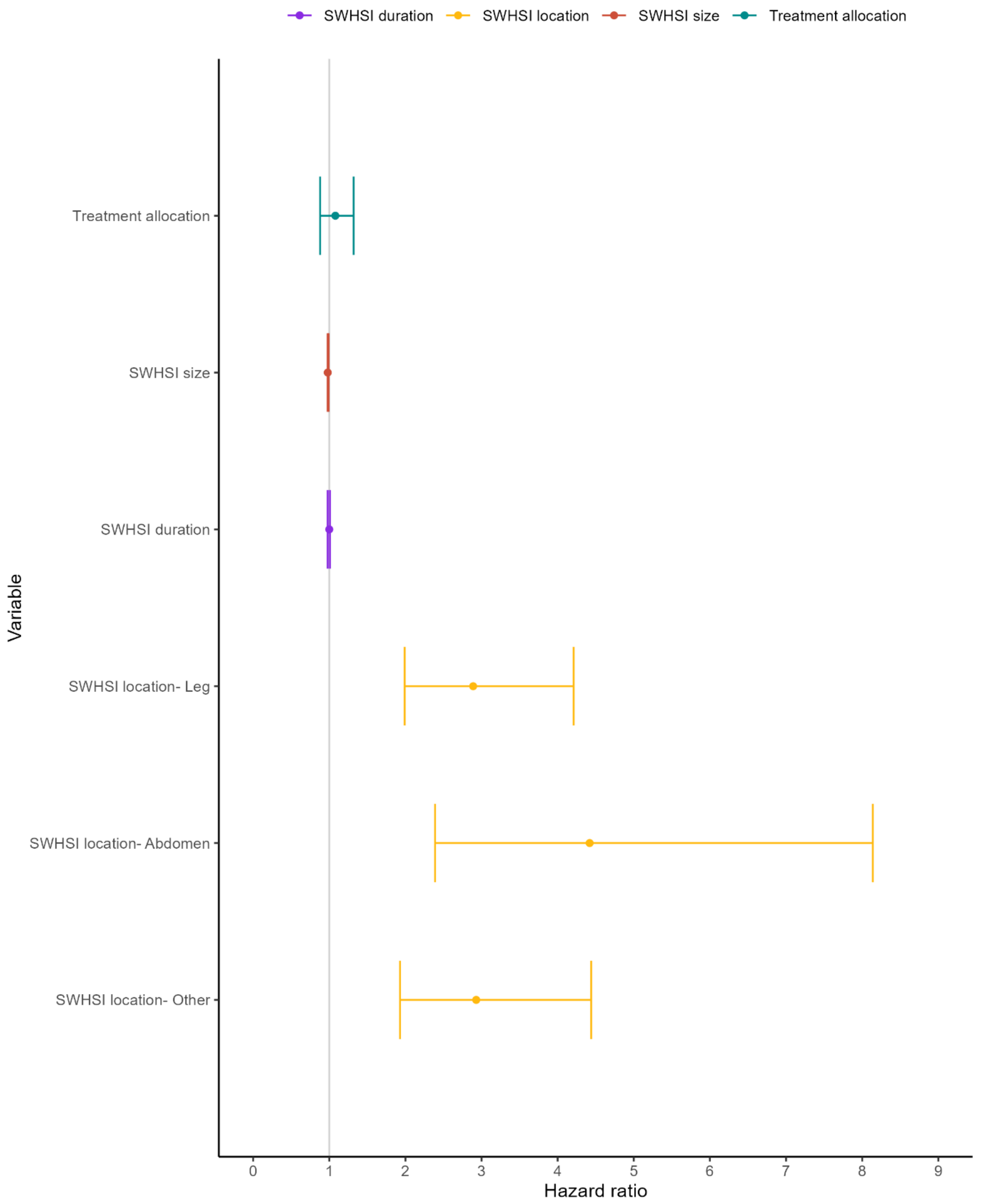

Figure 1: Forest plot by stratification variables (wound size, duration and location)

Subgroup analysis

Table 5: Subgroup analysis

	n	Hazard Ratio (95% CI)	p-value
Subgroup: Previous history of SWHSI*	641	1·00 (0·66 – 1·50)	0·98

*Treatment effect is the interaction term between allocation and history of SWHSI (yes/no)

Adverse Event data

Table 6: Summary of adverse events

	NPWT (N=232)	Usual care (N=197)
**AE Category**		
Allergic reaction (Usual Care)	0 (0·0%)	1 (0·5%)
Amputation (major)	14 (6·0%)	19 (9·6%)
Amputation (minor)	8 (3·4%)	3 (1·5%)
Angiogram	0 (0·0%)	1 (0·5%)
Angioplasty	1 (0·4%)	1 (0·5%)
Bruising	1 (0·4%)	0 (0·0%)
Death	1 (0·4%)	0 (0·0%)
Debridement	5 (2·2%)	2 (1·0%)
Delay in NPWT application	1 (0·4%)	0 (0·0%)
Hypergranulation	1 (0·4%)	0 (0·0%)
Increased Exudate	0 (0·0%)	2 (1·0%)
Ischaemia	2 (0·9%)	1 (0·5%)
Limb ischaemia	0 (0·0%)	1 (0·5%)
Necrosis	1 (0·4%)	1 (0·5%)
NPWT discomfort	2 (0·9%)	0 (0·0%)
NPWT failure	1 (0·4%)	1 (0·5%)
Overgranulation	1 (0·4%)	4 (2·0%)
Periwound Skin Change	5 (2·2%)	5 (2·5%)
Revascularisation – angioplasty	7 (3·0%)	6 (3·0%)
Revascularisation – angioplasty (failed)	0 (0·0%)	1 (0·5%)
Revascularisation – surgical (CFA endarterectomy)	1 (0·4%)	0 (0·0%)
Skin Damage (NPWT)	1 (0·4%)	0 (0·0%)
Surgical debridement	2 (0·9%)	1 (0·5%)
Wound bleeding	3 (1·3%)	0 (0·0%)
Wound breakdown	0 (0·0%)	1 (0·5%)
Wound closure – partial delayed surgical closure	0 (0·0%)	1 (0·5%)
Wound closure (skin graft)	1 (0·4%)	1 (0·5%)
Wound deterioration	0 (0·0%)	1 (0·5%)
Wound infection (major – debridement)	12 (5·2%)	10 (5·1%)

Wound Infection (major – debridement)	1 (0·4%)	1 (0·5%)
Wound infection (major – major amputation)	6 (2·6%)	4 (2·0%)
Wound Infection (major – major amputation)	1 (0·4%)	0 (0·0%)
Wound infection (major – minor amputation)	10 (4·3%)	5 (2·5%)
Wound infection (minor)	114 (49·1%)	88 (44·7%)
Wound infection (moderate – wound closure)	0 (0·0%)	1 (0·5%)
Wound infection (moderate)	29 (12·5%)	30 (15·2%)
Wound leakage	0 (0·0%)	1 (0·5%)
Wound not progressing	0 (0·0%)	2 (1·0%)
Wound pain	0 (0·0%)	1 (0·5%)
Not reported	0	0
**Relationship to Treatment**		
Not related	95 (40·9%)	92 (46·7%)
Unlikely to be related	78 (33·6%)	44 (22·3%)
Possibly related	26 (11·2%)	23 (11·7%)
Probably related	14 (6·0%)	17 (8·6%)
Definitely related	19 (8·2%)	21 (10·7%)
Not reported	0	0

Table 7: Summary of serious adverse events

	NPWT (n=9)	Usual care (n=5)
**SAE Category**		
Death	1 (11%)	0 (0%)
Medication overdose	0 (0%)	1 (20%)
Revascularisation - angioplasty	1 (11%)	1 (20%)
Wound bleeding	1 (11%)	0 (0%)
Wound infection (major - debridement)	4 (44%)	0 (0%)
Wound infection (Major - Endartectomy)	0 (0%)	1 (20%)
Wound infection (major - revascularisation)	0 (0%)	2 (40%)
Wound infection (moderate)	1 (11%)	0 (0%)
Wound infection (severe)	1 (11%)	0 (0%)
**Relationship to Treatment**		
Not related	2 (22%)	3 (60%)
Unlikely to be related	3 (33%)	2 (40%)
Possibly related	3 (33%)	0 (0%)

Missing data*	1 (11%)	0 (0%)

*Cause of death was unknown as GP service did not provide Medical Certificate of Cause of Death

Dressings used in each treatment group

Table 8: Summary of dressings used in each group (where provided)

**Dressings used**	
**NPWT group**	NPWT group (n=349)
Black, polyurethane foam dressings with reticulated (open) pores	193 (68%)
Antimicrobial gauze (impregnated with Polyhexamethylene biguanide)	69 (24%)
White, polyvinyl alcohol with high tensile strength, pre-moistened with sterile water	22 (8%)
**Usual care group**	Usual care group (n=337)
Alginate	87 (25·8%)
Hydrocolloid	49 (14·5%)
Superabsorbent	48 (14·2%)
Aquacel/Hydro fibre	45 (13·4%)
Basic wound contact	42 (12·5%)
Foam	37 (11%)
Silver containing	28 (8·3%)
Softban/Crepe bandage	24 (7·1%)
Soft polymer	17 (5%)
Gauze Ribbon	16 (4·7%)
Iodine Containing	14 (4·2%)
Adaptic touch/Sorbisan	14 (4·2%)
Flaminal	9 (2·7%)
Atrauman	8 (2·4%)
Hydrogel	7 (2·1%)
Hydrofoam/Spun Hydrocolloid	5 (1·5%)
Honey	3 (0·9%)
Protease Modulating matrix	3 (0·9%)
Flamazine silver sulfadiazine dressing	3 (0·9%)
PHMB	1 (0·3%)
Urgotol silver sulfadiazine dressing	1 (0·3%)
Vapour permeable film or membrane	1 (0·3%)
Other	47 (13·9%)

Amputations in secondary analysis by wound location

Amputation by wound location	NPWT (n=35)	Usual care (n=36)
Foot	30 (86%)	34 (94%)
Leg	5 (14%)	2 (6%)

1. Grambsch PM. and Therneau TM. Proportional hazards tests and diagnostics based on weighted residuals. *Biometrika* 1994; 81: 515-526. DOI: https://doi.org/10.1093/biomet/81.3.515.

Additional Analyses – Health Economics

Section A. Unit costs

Table i: Unit costs of primary and secondary healthcare resources

Health Resource	Unit cost	Description	Source
GP surgery appointment	£41.00	Based on £41/9.22 minutes. 9.22-minute appointment	PSSRU 2023
GP home appointment	£107.01	Based on £41/9.22 minutes. 11.4-minute home visit, 12 minutes of travel and 5 miles of travel assumed (£0.59 per mile)	PSSRU 2023
Nurse surgery appointment	£13.43	Based on £52/h and 15.5 minutes of patient contact.	PSSRU 2023
Nurse home appointment	£26.78	Based on minutes of travel and 5 miles of travel (£0.59/mile).	PSSRU 2023
Hospital outpatient appointment (Diabetic Foot Clinic)	£183.00	Service code: 307 (Diabetes Service)	NHS Reference Costs 2021/2022
Hospital outpatient appointment (Podiatry)	£93.00	Service code: 653 (Podiatry Service)	NHS Reference Costs 2021/2022
Hospital outpatient appointment (Specialty Dressing Clinic)	£175.00	Currency code: JB71A (Cleansing and Dressing of Burn, 16 years and over)	NHS Reference Costs 2021/2022
Hospital outpatient appointment (Vascular, Colorectal or Plastics)	£176.33	Average of service codes 104 (Colorectal Surgery Service), 107 (Vascular Surgery Service), 160 (Plastic Surgery Service)	NHS Reference Costs 2021/2022
Hospital admission without overnight	£1,038.36	Average cost of elective hospital stays.	NHS Reference Costs 2021/2022
Hospital inpatient nights (per night)	£492.19	Elective inpatient excess bed-days (£431). Inflation adjusted according to World Bank GDP deflator for UK.	NHS Reference Costs 2017/2018
Accident and emergency	£242.00	Emergency care	NHS Reference Costs 2021/2022

Table ii: Unit costs of medications

Medication	Unit cost (per day)	Description	Source
Paracetamol	£0.19	Paracetamol 500 mg caplets. 100 tablets: £2.34. Max use: 8 tablets per day.	BNF 2023
Co-codamol	£0.26	Co-codamol 8mg/500mg, 100 tablets: £3.27. Max use: 8 tablets per day.	BNF 2023
Tramadol	£1.07	Zydol 50mg soluble tablets, 100 tablets: £13.33 Max use: 8 tablets per day.	BNF 2023
Codeine Phosphate	£0.42	Codeine 15mg tablets, 100 tablets: £2.64. Max use: 16 tablets per day.	BNF 2023
Short acting morphine	£0.51	Morphine sulphate 20 mg tablets, 56 tablets: £9.50. Max use: Three 20 mg tablets per day.	BNF 2023
Long-acting morphine	£0.41	Morphine sulphate 60 mg tablets, 60 tablets: £24.32. Max use: One 60mg tablet per day.	BNF 2023
Other morphine-based tablets	£0.45	Kaolin and Morphine mixture; 200 ml: £2.27 Max use: 40 mL per day.	BNF 2023
Morphine oral suspension	£0.81	Morphine sulphate 10mg/5ml oral solution. 500 ml: £6.72. Max use: 120mg per day, i.e. 60ml.	BNF 2023
Amitriptyline	£0.07	Amitriptyline 25mg tablets. 28 tablets: £0.68. Max use: 75 mg per day (neuropathic pain), i.e. 3 tablets per day.	BNF 2023
Pregabalin	£0.34	Pregabalin 75mg tablets. 56 tablets: £4.79. Initially 150 mg daily in 2–3 divided doses, then increased if necessary to 300 mg daily in 2–3 divided doses, dose to be increased after 3–7 days, then increased, if necessary, up to 600 mg daily in 2–3 divided doses, dose to be increased after 7 days. Assume 300mg per day, i.e. 4 tablets per day.	BNF 2023
Gabapentin	£0.08	Gabapentin 300mg capsules. 100 capsules: £2.55. Max use: 3 capsules (900mg) per day.	BNF 2023
Diclofenac	£2.91	Diclofenac .74 mg per 1 ml. 200mL: £12.95. Max use: 45 mL per day.	BNF 2023
Aspirin	£1.63	Aspirin 300mg tablets. 100 tablets: £12.56. Max use: 4g per day for pyrexia (by mouth), i.e. 13 tablets	BNF 2023
Ibuprofen	£0.22	Ibuprofen 200mg tablets. 250 tablets: £9.30. Max use: 1200mg per day, i.e. 6 tablets per day.	BNF 2023
Antibiotics	£1.06	Weighted average of:<br>• Flucloxacillin 500mg capsules. 28 capsules: £2.01. Max use: 2g/day, i.e. 4 tablets a day (£0.29)<br>• Co-amoxiclav 500/125 mg capsules. 21 capsules. Max use: 500/125 mg every 8 hours for foot infection/leg ulcers, i.e. 3 tablets a day (£0.48)<br>• Metronidazole 400mg tablets. Max use: 400 mg every 8 hours for leg ulcers, i.e. 3 tablets a day (£0.15)<br>• Clindamycin 300 mg tablets. Max use: 300 mg every 6 hours for foot infection, i.e. 4 tablets a day (£4.60)	BNF 2023

		• Ciprofloxacin. 500mg tablets. Max use: 500 mg twice daily, i.e. 2 tablets a day (£0.20)<br>• Doxycycline. Max use: 100mg daily (200mg day 1 and then 100mg for another 6 days), i.e. 1 tablet a day on average (£0.61)	
Beta Blockers	£0.03	Amlodipine 5 mg 28 tablets. Max use: 5mg per day.	BNF 2023
Anticoagulant	£0.18	Apixaban 5mg 56 tablets. Max use: 10 mg twice daily, i.e. 2 tablets daily.	BNF 2023
Atorvastatin	£0.06	Atorvastatin 20mg 28 tablets Max use: 20 mg once daily.	BNF 2023
Clopidogrel	£0.04	Clopidogrel 75mg 28 tablets. Max use: 75 mg once daily.	BNF 2023
Analgesics (Oxycodone)	£8.94	Oxycodone modified released medicine 40 mg 56 tablets-. Max use: 400mg per day, i.e. 10 tablets per day.	BNF 2023
Metformin	£0.02	Metformin 28 tablets (500mg). Max use: 500mg per day.	BNF 2023
Omeprazole	£0.21	Omeprazole 28 tablets (20mg). Max use: 20mg per day.	
Duloxetine	£0.31	Duloxetine 28 tablets (60mg). Max dose: 60mg per day.	BNF 2023
Isosorbide Mononitrate	£0.73	Isosorbide Mononitrate 56 tablets (40mg). Max dose: 40mg twice per day.	BNF 2023
Perindopril	£0.04	Perindopril erbumine 30 tablets (4mg). Max dose: 4mg per day.	BNF 2023

Table iii: Intervention-related daily costs

**Standard dressings**		
**Type of dressing**	**Unit cost**	**Source**
Alginate dressing (advanced)	£2.46	BNF 2023
Hydrogel dressing (advanced)	£4.62	BNF 2023
Hydrocolloid dressing (advanced)	£3.37	BNF 2023
Basic wound contact dressing	£0.98	BNF 2023
Hydrofibre/spun Hydrocolloid dressing (advanced)	£4.19	BNF 2023
Foam dressing (advanced)	£4.13	BNF 2023
Soft Polymer dressing (advanced)	£5.55	BNF 2023
Cavity foam dressing	£3.49	BNF 2023
Silver containing	£10.79	BNF 2023
Iodine containing	£3.28	BNF 2023
Superabsorbant dressing (advanced)	£4.97	BNF 2023
Gauze Ribbon	£0.00	BNF 2023
Honey	£3.98	BNF 2023
PHMB	£7.24	BNF 2023
Protease Modulating Matrix	£6.16	BNF 2023
Vapour permeable	£1.51	BNF 2023
**Average daily cost of standard dressings (SE)**	**£4.17 (£0.11)**	
**NPWT**		
**Type of device**	**Unit cost**	**Source**

PICO7	£15.43	Data from Cornwall site.
**Type of dressing**	**Unit cost**	**Source**
Medium dressing (Granufoam)	£11.03	Data from Cornwall site.
Small dressing (Granufoam)	£9.48	Data from Cornwall site.
White foam	£10.16	Data from Cornwall site.
Gauze	£13.01	Data from Cornwall site.
Small canister/ Small foam dressing	£16.65	Data from Hull site.
Small canister/ Medium foam dressing	£17.21	Data from Hull site.
Small canister/ Large foam dressing	£17.89	Data from Hull site.
Small canister/ Gauze dressing	£13.71	Data from Hull site.
Small canister /Polyvinyl dressing	£14.19	Data from Hull site.
Medium canister/ Small foam dressing	£18.98	Data from Hull site.
Medium canister/ Medium foam dressing	£19.54	Data from Hull site.
Medium canister/ Large foam dressing	£20.22	Data from Hull site.
Medium canister/ Gauze dressing	£16.04	Data from Hull site.
Medium canister /Polyvinyl dressing	£16.52	Data from Hull site.
Average daily cost of dressings	*£15.18*	
Average daily cost of device	*£15.43*	
**Average daily cost of NPWT (SE)**	**£30.61 (£3.41)**	

Note: The average daily costs of dressings for both standard dressings and NPWT were based on the average of the presented daily unit costs of different types of dressings. Regarding NPWT, the average daily cost of the device is also incorporated to estimate its average daily cost. The presented costs aimed to cover the most realistic practices of delivering NPWT and standard dressings and thus to produce the most accurate daily costs of these treatments given the information available.

Section B. Accounting for missing data

Mean imputation was performed on missing data related to baseline variables, including baseline EQ-5D-5L scores and baseline costs, as these were assumed to be treatment independent. For missing data on follow-up variables, we applied multiple imputation using predictive mean matching, by considering the 10 closest neighbouring values and assuming data are missing at random (MAR), i.e. that the likelihood of data missingness is related to observed rather than unobserved data. The number of imputations, set to 50, was determined according to the highest percentage of missing data (see *Table iv*). The multiple imputation model included baseline utilities, baseline costs, and other baseline variables found to be significantly correlated with data missingness, according to logistic regressions of missing QALYs and missing costs (see *Table v*). The multiply imputed data on EQ-5D scores and costs were estimated using Rubin's rules (*mi estimate* command in STATA). Missing data on resource use and EQ-5D-5L scores were set to zero, after the date of death, for participants reported to have died during the trial's follow-up period of 12 months. Multiple imputation was applied to the base-case cost-utility analysis, as well as to the sensitivity analyses, and was undertaken through Stata 18.

Table iv: Missing data table, by group and total

	NPWT (n=349)		Usual care (n=337)		Total (n=686)	
**Variables**	**n of missing**	**% of missing**	**n of missing**	**% of missing**	**n of missing**	**% of missing**
**Baseline**						
Age	3	0.9%	1	0.3%	4	0.6%
Gender	3	0.9%	0	0.0%	3	0.4%
EQ-5D-5L score	14	4.0%	16	4.7%	30	4.4%
Duration of SWHSI	3	0.9%	0	0.0%	3	0.4%
Number of SWHSI	3	0.9%	0	0.0%	3	0.4%
Location of SWHSI	0	0.0%	0	0.0%	0	0.0%
Area of SWHSI	15	4.3%	4	0.6%	19	2.8%
Treatment location	4	1.1%	0	0.0%	4	0.6%
History of previous SWHSI	26	7.4%	19	5.6%	45	6.6%
Infection of SWHSI	3	0.9%	1	0.3%	4	0.6%
Full thickness skin loss	3	0.9%	0	0.0%	3	0.4%
Subcutaneous issue exposed	3	0.9%	0	0.0%	3	0.4%
Underlying muscle exposed	3	0.9%	0	0.0%	3	0.4%
Underlying tendons exposed	3	0.9%	0	0.0%	3	0.4%
Underlying bone exposed	3	0.9%	0	0.0%	3	0.4%
Tissue involvement unclear due to devitalised tissue	3	0.9%	0	0.0%	3	0.4%
**3 months**						
EQ-5D-5L score	140	40.1%	109	32.3%	249	36.3%
GP surgery visits	145	41.5%	127	37.7%	272	39.7%
GP home visits	139	39.8%	121	35.9%	260	37.9%
Nurse surgery visits	152	43.6%	138	40.9%	290	42.3%
Nurse home visits	162	46.4%	148	43.9%	310	45.2%
Secondary care use	29	8.3%	18	5.3%	47	6.9%
Medication use	176	50.4%	171	50.7%	347	50.6%
**6 months**						
EQ-5D-5L score	127	36.4%	127	37.7%	254	37.0%
GP surgery visits	142	40.7%	155	46.0%	297	43.3%
GP home visits	140	40.1%	150	44.5%	290	42.3%
Nurse surgery visits	139	39.8%	158	46.9%	297	43.3%
Nurse home visits	151	43.3%	160	47.5%	311	45.3%
Secondary care use	29	8.3%	18	5.3%	47	6.9%
Medication use	176	50.4%	171	50.7%	347	50.6%
**12 months**						
EQ-5D-5L score	126	36.1%	130	38.6%	256	37.3%
GP surgery visits	161	46.1%	166	49.3%	327	47.7%
GP home visits	162	46.4%	163	48.4%	325	47.4%
Nurse surgery visits	166	47.6%	167	49.6%	333	48.5%
Nurse home visits	168	48.1%	168	49.9%	336	49.0%
Secondary care use	29	8.3%	18	5.3%	47	6.9%

Medication use	176	50.4%	171	50.7%	347	50.6%

Table v: Logistic regressions (correlation between missing QALYs/missing costs and baseline variables)

	**Odds ratio in logistic regression for missing data (95% confidence interval; p-value)**	
	**Missing data on QALYs**	**Missing data on costs**
Treatment allocation	1.04 [(95% CI: 0.76 to 1.42); (p-value= 0.82)]	1.24 [(95% CI: 0.87 to 1.76); (p-value= 0.24)]
Age	0.97 [(95% CI: 0.96 to 0.98); (p-value= 0.00)]*	0.99 [(95% CI: 0.97 to 1.00); (p-value= 0.11)]
Gender	0.83 [(95% CI: 0.56 to 1.22); (p-value= 0.34)]	0.74 [(95% CI: 0.47 to 1.16); (p-value= 0.19)]
EQ-5D-5L score (baseline)	0.48 [(95% CI: 0.25 to 0.89); (p-value= 0.02)]*	0.36 [(95% CI: 0.17 to 0.74); (p-value= 0.01)]*
Duration of SWHSI	1.00 [(95% CI: 0.97 to 1.02); (p-value= 0.75)]	1.04 [(95% CI: 1.00 to 1.08); (p-value= 0.04)]
Number of SWHSI	1.06 [(95% CI: 0.71 to 1.60); (p-value= 0.77)]	0.83 [(95% CI: 0.53 to 1.32); (p-value= 0.44)]
Location of SWHSI	0.92 [(95% CI: 0.73 to 1.17); (p-value= 0.50)]	0.93 [(95% CI: 0.71 to 1.20); (p-value= 0.56)]
Area of SWHSI	0.99 [(95% CI: 0.99 to 1.00); (p-value= 0.05)]*	1.00 [(95% CI: 1.00 to 1.01); (p-value= 0.78)]
Treatment location	0.94 [(95% CI: 0.85 to 1.04); (p-value= 0.20)]	1.04 [(95% CI: 0.91 to 1.17); (p-value= 0.58)]
History of previous SWHSI	1.20 [(95% CI: 0.76 to 1.89); (p-value= 0.42)]	1.69 [(95% CI: 0.98 to 2.92); (p-value= 0.06)]**
Infection of SWHSI	1.10 [(95% CI: 0.68 to 1.79); (p-value= 0.70)]	0.70 [(95% CI: 0.41 to 1.18); (p-value= 0.18)]
Full thickness skin loss	0.71 [(95% CI: 0.50 to 1.01); (p-value= 0.06)]**	0.53 [(95% CI: 0.36 to 0.80); (p-value= 0.00)]*
Subcutaneous issue exposed	1.29 [(95% CI: 0.91 to 1.82); (p-value= 0.15)]	1.31 [(95% CI: 0.89 to 1.92); (p-value= 0.18)]
Underlying muscle exposed	1.14 [(95% CI: 0.78 to 1.66); (p-value= 0.51)]	1.04 [(95% CI: 0.69 to 1.58); (p-value= 0.85)]
Underlying tendons exposed	1.09 [(95% CI: 0.72 to 1.65); (p-value= 0.70)]	1.24 [(95% CI: 0.77 to 1.99); (p-value= 0.39)]
Underlying bone exposed	0.86 [(95% CI: 0.56 to 1.31); (p-value= 0.48)]	1.47 [(95% CI: 0.89 to 2.42); (p-value= 0.14)]
Tissue involvement unclear due to devitalised tissue	0.68 [(95% CI: 0.17 to 2.67); (p-value= 0.58)]	0.66 [(95% CI: 0.15 to 2.87); (p-value= 0.58)]

* if significant at the 5% level

** if significant at the 10% level

Section C. Quality-Adjusted Life Years (QALYs), resource use and costs, based on all available cases

Table vi: Summary of EQ-5D-5L utility scores and mean difference (95% CI) at each time point, based on all available cases

	**NPWT (n=349)**		**Usual care (n=337)**		**Total (n=686)**		**Mean difference (unadjusted)**
**Health Utility**	**Mean (SE)**	**n (% missing)**	**Mean (SE)**	**n (% missing)**	**Mean (SE)**	**n (% missing)**	
EQ-5D-5L score (baseline)	0.614 (0.015)	335 (4.0%)	0.601 (0.016)	321 (4.7%)	0.607 (0.011)	656 (4.4%)	0.012 (95% CI: -0.030 to 0.055)
EQ-5D-5L score (3 months)	0.598 (0.022)	209 (40.1%)	0.581 (0.021)	228 (32.3%)	0.589 (0.015)	437 (36.3 %)	0.017 (95% CI: -0.043 to 0.077)
EQ-5D-5L score (6 months)	0.590 (0.022)	222 (36.4%)	0.572 (0.024)	210 (37.7%)	0.581 (0.016)	432 (37.0 %)	0.019 (95% CI: -0.045 to 0.083)
EQ-5D-5L score (12 months)	0.524 (0.025)	223 (36.1%)	0.514 (0.025)	207 (38.6%)	0.519 (0.017)	430 (37.3 %)	0.010 (95% CI: --0.059 to 0.078)
QALYs (all available)	*0.575 (0.024)*	*151 (56.7%)*	*0.566 (0.024)*	*143 (57.6%)*	*0.571 (0.017)*	*294 (57.1 %)*	*0.009 (95% CI: -0.058 to 0.076)*

Note: The QALYs were estimated using the area under the curve (AUC) approach.

Table vii: Mean resource use of primary and secondary healthcare services

	**NPWT (n=349)**		**Usual care (n=337)**		**Total (n=686)**	
**Resource item**	**Mean (SD)**	**n (%missing)**	**Mean (SD)**	**n (% missing)**	**Mean (SD)**	**n (% missing)**
GP surgery appointment						
Baseline	0.214 (0.777)	337 (3.4%)	0.218 (0.891)	325 (3.6%)	0.216 (0.834)	662 (3.5%)
3 months	0.116 (0.726)	204 (41.5%)	0.178 (0.861)	210 (37.7%)	0.147 (0.797)	414 (39.7%)
6 months	0.037 (0.198)	207 (40.7%)	0.324 (2.490)	182 (46.0%)	0.171 (1.713)	389 (43.3%)
12 months	0.043 (0.182)	188 (46.1%)	0.055 (0.346)	171 (49.3%)	0.048 (0.272)	359 (47.7%)
GP home appointment						
Baseline	0.029 (0.185)	342 (2.0%)	0.040 (0.263)	325 (3.6%)	0.034 (0.227)	667 (2.8%)
3 months	0.048 (0.203)	210 (39.8%)	0.082 (0.604)	216 (35.9%)	0.065 (0.453)	426 (37.9%)

6 months	0.021 (0.147)	209 (40.1%)	0.018 (0.108)	187 (44.5%)	0.019 (0.130)	396 (42.3%)
12 months	0.004 (0.030)	187 (46.4%)	0.011 (0.105)	174 (48.4%)	0.007 (0.076)	361 (47.4%)
Nurse surgery appointment						
Baseline	0.483 (2.149)	344 (1.4%)	0.530 (2.404)	328 (2.7%)	0.506 (2.275)	672 (2.0%)
3 months	1.306 (3.457)	197 (43.6%)	1.258 (2.829)	199 (40.9%)	1.282 (3.153)	396 (42.3%)
6 months	0.570 (1.921)	210 (39.8%)	0.741 (2.605)	179 (46.9%)	0.649 (2.260)	389 (43.3%)
12 months	0.404 (1.952)	183 (47.6%)	0.519 (1.795)	170 (49.6%)	0.459 (1.876)	353 (48.5%)
Nurse home appointment						
Baseline	1.122 (4.106)	337 (3.4%)	1.656 (7.878)	320 (5.0%)	1.382 (6.236)	657 (4.2%)
3 months	4.465 (5.919)	187 (46.4%)	4.365 (6.761)	189 (43.9%)	4.415 (6.348)	376 (45.2%)
6 months	1.463 (4.273)	198 (43.3%)	1.218 (3.638)	177 (47.5%)	1.348 (3.983)	375 (45.3%)
12 months	0.459 (2.700)	181 (48.1%)	0.345 (1.737)	169 (49.9%)	0.404 (2.284)	350 (49.0%)
Hospital outpatient appointment (Diabetic Foot Clinic)						
Baseline	0.336 (1.074)	339 (2.9%)	0.355 (1.013)	327 (3.0%)	0.345 (1.044)	666 (2.9%)
3 months	0.317 (0.658)	320 (8.3%)	0.372 (0.702)	319 (5.3%)	0.344 (0.680)	639 (6.9%)
6 months	0.078 (0.244)	320 (8.3%)	0.092 (0.260)	319 (5.3%)	0.085 (0.252)	639 (6.9%)
12 months	0.015 (0.086)	320 (8.3%)	0.016 (0.094)	319 (5.3%)	0.015 (0.090)	639 (6.9%)
Hospital outpatient appointment (Podiatry)						
Baseline	NA	NA	NA	NA	NA	NA
3 months	0.088 (0.359)	320 (8.3%)	0.069 (0.289)	319 (5.3%)	0.078 (0.326)	639 (6.9%)
6 months	0.025 (0.132)	320 (8.3%)	0.015 (0.101)	319 (5.3%)	0.020 (0.118)	639 (6.9%)
12 months	0.002 (0.019)	320 (8.3%)	0.002 (0.023)	319 (5.3%)	0.002 (0.021)	639 (6.9%)
Hospital outpatient appointment (Specialty Dressing Clinic)						
Baseline	0.143 (1.092)	343 (1.7%)	0.134 (1.139)	328 (2.7%)	0.139 (1.114)	671 (2.2%)
3 months	0.021 (0.138)	320 (8.3%)	0.025 (0.150)	319 (5.3%)	0.023 (0.144)	639 (6.9%)
6 months	0.000 (0.000)	320 (8.3%)	0.008 (0.095)	319 (5.3%)	0.004 (0.067)	639 (6.9%)
12 months	0.001 (0.009)	320 (8.3%)	0.001 (0.009)	319 (5.3%)	0.001 (0.009)	639 (6.9%)
Hospital outpatient appointment (Vascular, Colorectal or Plastics)						
Baseline	0.157 (0.615)	343 (1.7%)	0.151 (0.661)	325 (3.6%)	0.154 (0.638)	668 (2.6%)
3 months	0.048 (0.126)	320 (8.3%)	0.047 (0.143)	319 (5.3%)	0.047 (0.135)	639 (6.9%)
6 months	0.016	320	0.015	319	0.015	639

	(0.080)	(8.3%)	(0.086)	(5.3%)	(0.083)	(6.9%)
12 months	0.005 (0.033)	320 (8.3%)	0.005 (0.036)	319 (5.3%)	0.005 (0.035)	639 (6.9%)
Hospital admission without overnight						
Baseline	NA	NA	NA	NA	NA	NA
3 months	0.004 (0.037)	320 (8.3%)	0.003 (0.032)	319 (5.3%)	0.004 (0.035)	639 (6.9%)
6 months	0.000 (0.000)	320 (8.3%)	0.005 (0.049)	319 (5.3%)	0.003 (0.035)	639 (6.9%)
12 months	0.001 (0.013)	320 (8.3%)	0.001 (0.009)	319 (5.3%)	0.001 (0.011)	639 (6.9%)
Hospital inpatient nights (per night)						
Baseline	1.346 (4.900)	344 (1.4%)	1.061 (4.419)	328 (2.7%)	1.207 (4.670)	672 (2.0%)
3 months	1.013 (3.565)	320 (8.3%)	1.036 (3.980)	319 (5.3%)	1.024 (3.775)	639 (6.9%)
6 months	0.465 (2.874)	320 (8.3%)	0.724 (3.625)	319 (5.3%)	0.594 (3.271)	639 (6.9%)
12 months	0.255 (2.396)	320 (8.3%)	0.346 (2.925)	319 (5.3%)	0.300 (2.671)	639 (6.9%)
Accident and emergency						
Baseline	0.125 (0.451)	344 (1.4%)	0.113 (0.544)	328 (2.7%)	0.119 (0.498)	672 (2.0%)
3 months	0.022 (0.091)	320 (8.3%)	0.036 (0.180)	319 (5.3%)	0.029 (0.142)	639 (6.9%)
6 months	0.004 (0.037)	320 (8.3%)	0.010 (0.064)	319 (5.3%)	0.007 (0.052)	639 (6.9%)
12 months	0.006 (0.036)	320 (8.3%)	0.005 (0.035)	319 (5.3%)	0.005 (0.035)	639 (6.9%)

Table viii: Total mean costs based on all available cases, up to 12-month follow-up

	**NPWT (n=349)**		**Usual care (n=337)**		**Total (n=686)**		**Mean difference (unadjusted)**
**Cost item**	**Mean (SE)**	**n (% missing)**	**Mean (SE)**	**n (% missing)**	**Mean (SE)**	**n (% missing)**	
GP surgery appointment	£29.87 (£10.59)	140 (59.9%)	£62.33 (£23.04)	123 (63.5%)	£45.05 (£12.17)	263 (61.7%)	-£32.46 (95% CI: -£80.44 to £15.51)
GP home appointment	£18.58 (£6.18)	144 (58.7%)	£39.72 (£20.92)	132 (60.8%)	£28.69 (£10.51)	276 (59.8%)	-£21.15 (95% CI: -£62.57 to £20.27)
Nurse surgery appointment	£122.56 (£23.69)	135 (61.3%)	£132.53 (£23.75)	114 (66.2%)	£127.13 (£16.80)	249 (63.7%)	-£9.97 (95% CI: -£76.51 to £56.57)
Nurse home appointment	£533.62 (£74.63)	122 (65.0%)	£458.98 (£65.82)	108 (68.0%)	£498.57 (£50.17)	230 (66.5%)	£74.64 (95% CI: -£123.65 to £272.94)
Hospital outpatient	£232.7	320	£272.49	319	£252.59	639	-£39.74

appointment (Diabetic Foot Clinic)	5 (£24.13)	(8.3%)	(£25.21)	(5.3%)	(£17.45)	(6.9%)	(95% CI: -£108.27 to £28.79)
Hospital outpatient appointment (Podiatry)	£32.55 (£6.72)	320 (8.3%)	£24.49 (£5.16)	319 (5.3%)	£28.53 (£4.24)	639 (6.9%)	£8.06 (95% CI: -£8.58 to £24.70)
Hospital outpatient appointment (Specialty Dressing Clinic)	£11.48 (£4.08)	320 (8.3%)	£18.10 (£6.21)	319 (5.3%)	£14.79 (£3.71)	639 (6.9%)	-£6.62 (95% CI: -£21.20 to £7.96)
Hospital outpatient appointment (Vascular, Colorectal or Plastics)	£38.57 (£5.20)	320 (8.3%)	£37.59 (£7.04)	319 (5.3%)	£38.08 (£4.37)	639 (6.9%)	£0.98 (95% CI: -£16.18 to £18.15)
Hospital admission without overnight	£19.47 (£7.89)	320 (8.3%)	£29.30 (£10.69)	319 (5.3%)	£24.37 (£6.64)	639 (6.9%)	-£9.83 (95% CI: -£35.90 to £16.24)
Hospital inpatient nights (per night)	£2934.01 (£824.34)	320 (8.3%)	£3621.22 (£964.58)	319 (5.3%)	£3277.08 (£633.91)	639 (6.9%)	-£687.21 (95% CI: -£3178.22 to £1803.80)
Accident and emergency	£27.23 (£5.13)	320 (8.3%)	£40.97 (£8.76)	319 (5.3%)	£34.08 (£5.08)	639 (6.9%)	-£13.74 (95% CI: -£33.66 to £6.18)
Medication costs	£90.05 (£13.94)	173 (50.4%)	£102.13 (£28.36)	166 (50.7%)	£95.96 (£15.58)	339 (50.6%)	-£12.08 (95% CI: -£73.46 to £49.30)
Intervention costs	£1700.86 (£106.22)	349 (0.0%)	£967.26 (£69.77)	337 (0.0%)	£1340.48 (£65.46)	686 (0.0%)	£733.59 (95% CI: £482.27 to £984.92)
Total costs	£4077.81 (£401.89)	104 (70.2%)	£6246.09 (£2347.22)	83 (75.4%)	£5040.20 (£1064.92)	187 (72.7%)	-£2168.29 (95% CI: -£6396.65 to £2060.08)

Section D. Quality-Adjusted Life Years (QALYs) and costs, based on multiply imputed data set

Table ix: Summary of EQ-5D-5L utility scores and mean difference (95% CI) at each time point, based on multiply imputed data set

	**NPWT (n=349)**	**Usual care (n=337)**	**Total (n=686)**	**Mean difference (unadjusted)**
**Health Utility**	**Mean (SE)**	**Mean (SE)**	**Mean (SE)**	
EQ-5D-5L score (baseline)	0.613 (0.014)	0.601 (0.015)	0.607 (0.010)	0.012 (95% CI: -0.029 to 0.052)
EQ-5D-5L score (3 months)	0.590 (0.019)	0.563 (0.020)	0.577 (0.014)	0.027 (95% CI: -0.027 to

				0.082)
EQ-5D-5L score (6 months)	0.567 (0.021)	0.563 (0.020)	0.579 (0.014)	0.022 (95% CI: -0.036 to 0.081)
EQ-5D-5L score (12 months)	0.541 (0.022)	0.532 (0.023)	0.536 (0.016)	0.009 (95% CI: -0.055 to 0.073)
QALYs	*0.581 (0.016)*	*0.562 (0.017)*	*0.571 (0.012)*	*0.019 (95% CI: -0.027 to 0.065)*

Note: The QALYs were estimated using the area under the curve (AUC) approach.

Table x: Total mean costs of health resources based on multiply imputed data set, up to 12-month follow-up

	**NPWT (n=349)**	**Usual care (n=337)**	**Total (n=686)**	**Mean difference (unadjusted)**
**Cost item**	**Mean (SE)**	**Mean (SE)**	**Mean (SE)**	
GP surgery appointment	£35.88 (£11.71)	£48.23 (£13.58)	£41.95 (£8.88)	-£12.36 (95% CI: -£47.92 to £23.20)
GP home appointment	£22.60 (£10.46)	£36.25 (£15.07)	£29.30 (£9.07)	-£13.65 (95% CI: -£49.89 to £22.59)
Nurse surgery appointment	£115.95 (£19.34)	£121.42 (£19.32)	£118.64 (£14.02)	-£5.46 (95% CI: -£58.13 to £47.20)
Nurse home appointment	£499.21 (£56.00)	£483.09 (£58.15)	£491.29 (£39.53)	£16.12 (95% CI: -£146.99 to £179.24)
Hospital outpatient appointment (Diabetic Foot Clinic)	£235.50 (£24.39)	£270.06 (£25.24)	£252.48 (£17.68)	-£34.56 (95% CI: -£102.90 to £33.78)
Hospital outpatient appointment (Podiatry)	£32.68 (£6.61)	£25.21 (£5.33)	£29.01 (£4.26)	£7.46 (95% CI: -£9.26 to £24.19)
Hospital outpatient appointment (Specialty Dressing Clinic)	£11.30 (£4.07)	£17.62 (£6.03)	£14.41 (£3.62)	-£6.32 (95% CI: -£20.48 to £7.84)
Hospital outpatient appointment (Vascular, Colorectal or Plastics)	£38.51 (£5.35)	£36.93 (£6.87)	£37.73 (£4.34)	£1.58 (95% CI: -£15.42 to £18.58)
Hospital admission without overnight	£19.04 (£7.69)	£28.72 (£10.41)	£23.79 (£6.43)	-£9.68 (95% CI: -£34.96 to £15.61)
Hospital inpatient nights (per night)	£2947.52 (£821.28)	£3618.22 (£954.87)	£3277.00 (£629.32)	-£670.70 (95% CI: -£3132.41 to £1791.01)
Accident and emergency	£27.17 (£5.15)	£41.08 (£8.65)	£34.00 (£5.03)	-£13.91 (95% CI: -£33.37 to

				£5.55)
Medication costs	£95.93 (£19.46)	£107.90 (£25.44)	£101.81 (£16.48)	-£11.97 (95% CI: -£72.83 to £48.88)
Intervention costs	£1700.86 (£106.22)	£967.26 (£69.77)	£1340.48 (£65.46)	£733.59 (95% CI: £482.27 to £984.92)
Total costs	£5782.14 (£830.36)	£5801.99 (£969.99)	£5791.89 (£637.49)	-£19.85 (95% CI: -£2516.81 to £2477.11)

Section E. Main cost-utility results

The within-trial economic analysis estimated the unadjusted mean per participant QALYs and mean per participant costs as 0.581 (95% CI: 0.549 to 0.612) and £5,782.13 (95% CI: £4,151.32 to £7,412.95) for the NPWT group and 0.562 (95% CI: 0.528 to 0.595) and £5,801.99 (95% CI: £3,897.19 to £7,706.78) for the usual care group. The mean difference-adjusted incremental QALYs and incremental costs suggest that NPWT increased QALYs by 0.007 (95% CI: -0.024 to 0.038) and increased costs by £ 251.44 (95% CI: £-2,192.63 to £2,695.52) over the 12-month study period. A cost-effectiveness plane (*Figure 1*) and a CEAC (*Figure 2*) present graphically the results of the base-case analysis.

Table xi: Cost-utility analysis (base-case analysis)

	**NPWT (n=349)**	**Usual care (n=337)**
Total QALYs	0.581 (95% CI: 0.549 to 0.612)	0.562 (95% CI: 0.528 to 0.595)
Incremental QALYs (adjusted) *	0.007 (95% CI: -0.024 to 0.038)	
Total costs	£5782.13 (95% CI: £4151.32 to £7412.95)	£5801.99 (95% CI: £3897.19 to £7706.78)
Incremental costs (adjusted) *	£ 251.44 (95% CI: -£2192.63 to £2695.52)	
**Cost-utility results**	**£20,000 cost-effectiveness threshold**	**£30,000 cost-effectiveness threshold**
Incremental net monetary benefit	-£93.22 (95% CI: -£119.16, -£67.28)	-£18.65 (95% CI: -£45.85, £8.55)
Probability of NPWT being cost-effective	47.2%	49.7%

*Seemingly unrelated regression

Figure 1: Cost-effectiveness plane (base-case analysis)

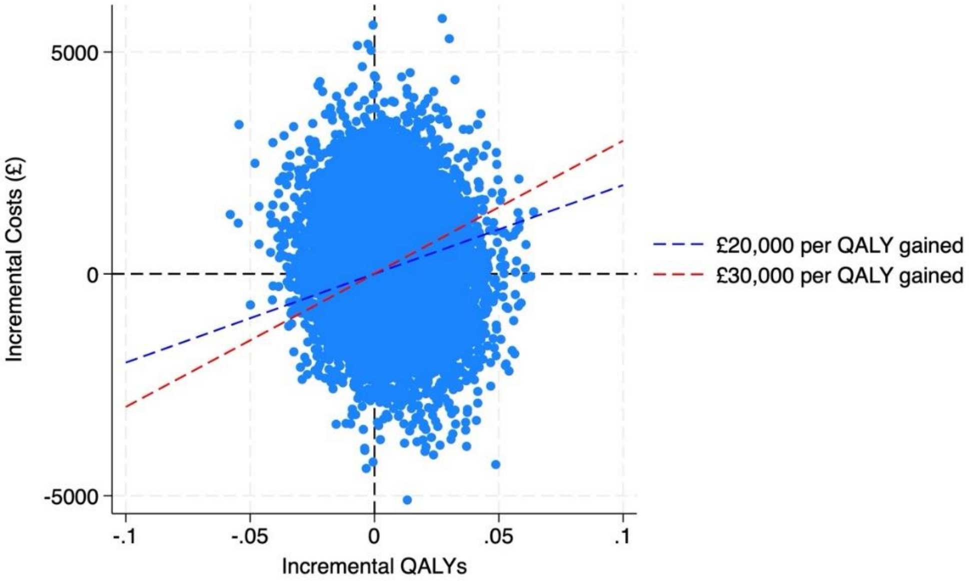

Figure 2: Cost-effectiveness acceptability curve (base-case analysis)

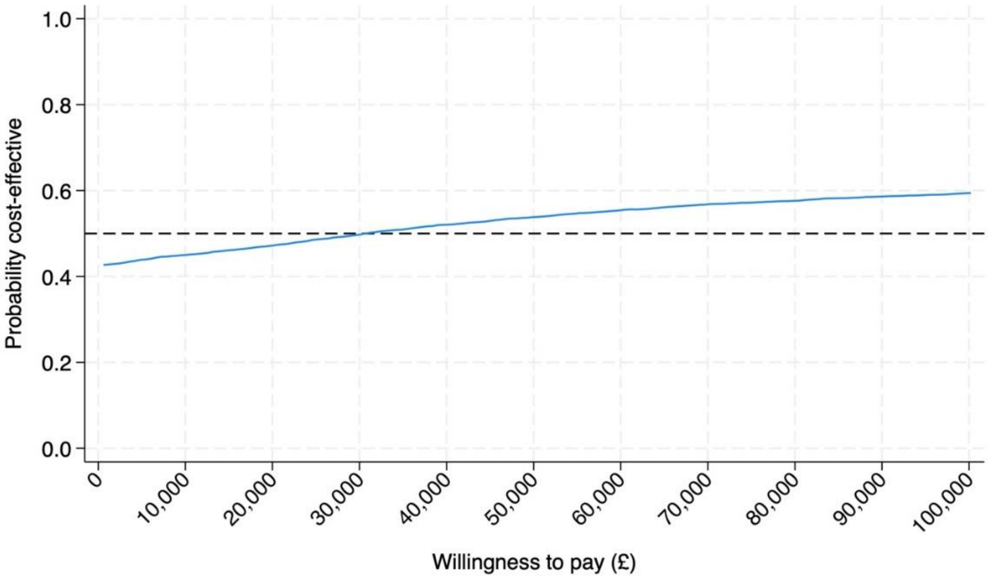

<u>Section F. Sensitivity analysis</u>

<u>*Complete-case analysis*</u>

Complete-case data were available for 167 participants (96 in the NPWT group and 71 in the usual care group). The complete-case analysis estimated the unadjusted mean per participant QALYs and mean per participant costs as 0.669 (95% CI: 0.623 to 0.715) and £4,019.35 (95% CI: £ 3,237.10 to £4,801.59) for the NPWT group and 0.678 (95% CI: 0.624 to 0 732) and £ 6,579.99 (95% CI: £1,128.59 to £12,031.39) for the usual care group. The mean difference-adjusted incremental QALYs and incremental costs suggest that NPWT reduced QALYs by -

0.000 (95% CI: -0.059 to 0.058) and reduced costs by -£1983.58 (95% CI: -£6868.42 to £2901.24) over the 12-month study period, although these results were not statistically significant. The INMB is £1,956.77 and £1,952.20 at the £20,000 and £30,000 cost-effectiveness thresholds respectively. The probability of cost-effectiveness of NPWT is 75.6% at the £20,000 threshold, and 74.6% at the £30,000 threshold. A cost-effectiveness plane and a CEAC present graphically these results (*Figure 3* and *Figure 4*).

Table xii: Cost-utility analysis (complete-case analysis)

**Complete-case analysis (Intention to treat)**		
	**NPWT (n=96)**	**Usual care (n=71)**
Total QALYs	0.669<br>(95% CI: 0.623 to 0.715)	0.678<br>(95% CI: 0.624 to 0 732)
Incremental QALYs (adjusted)*	-0.000 (95% CI: -0.059 to 0.058)	
Total costs	£4019.35<br>(95% CI: £ 3237.10 to £4801.59)	£ 6579.99<br>(95% CI: £1128.59 to £12031.39)
Incremental costs (adjusted)*	-£1983.58 (95% CI: -£6868.42 to £2901.24)	
**Cost-utility results**	**£20,000 cost-effectiveness threshold**	**£30,000 cost-effectiveness threshold**
Incremental net monetary benefit	£1956.77	£1952.20
Probability of NPWT being cost-effective	75.6%	74.6%

*Seemingly unrelated regressions

Figure 3: Cost-effectiveness plane (complete-case analysis)

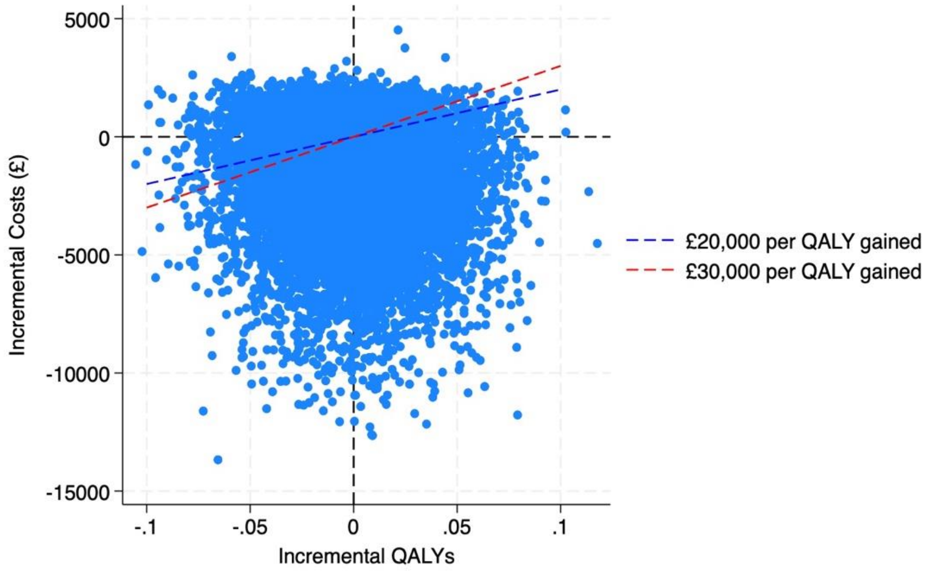

Figure 4: Cost-effectiveness acceptability curve (complete-case analysis)

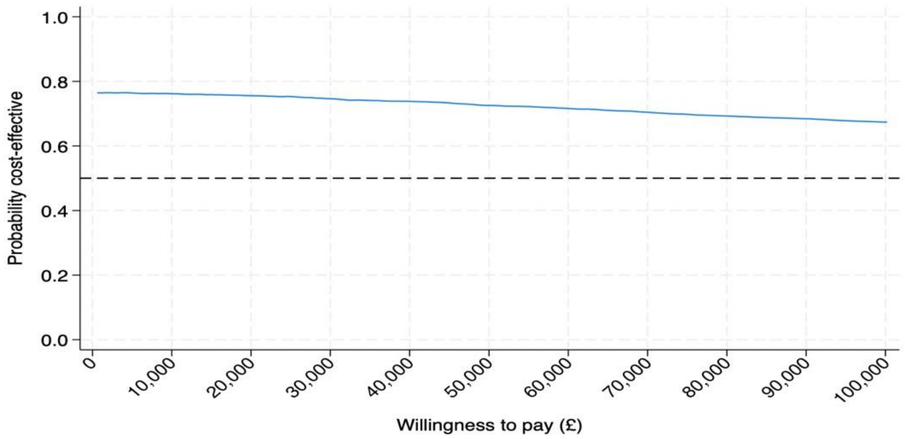

__Deaths excluded__

By excluding participants who died during the trial from the cost-utility analysis, the unadjusted mean per participant QALYs and mean per participant costs are 0.664 (95% CI: 0.614 to 0.675) and £ 6,173.83 (95% CI: £4,231.94 to £8,115.73) for the NPWT group and 0.634 (95% CI: 0.604 to 0.665) and £5,610.79 (95% CI: £3,652.69 to £7,568.88) for the usual care group. The mean difference-adjusted incremental QALYs and incremental costs suggest that NPWT increased QALYs by 0.002 (95% CI: -0.026 to 0.029) and increased costs by £987.37 (95% CI: -£1,558.27 to £3,533.02) over the 12-month study period, although these results were not statistically significant. The INMB is -£961.27 and -£942.45 at the £20,000 and £30,000 cost-effectiveness thresholds respectively. The probability of cost-effectiveness of NPWT is 24.2% at the £20,000 threshold, and 25.5% at the £30,000 threshold. A cost-effectiveness plane and a CEAC present graphically these results (*Figure 5* and *Figure 6*).

Table xiii: Cost-utility analysis (deaths excluded)

**Deaths excluded (Intention to treat)**		
	**NPWT (n=307)**	**Usual care (n=295)**
Total QALYs	0.664<br>(95% CI: 0.614 to 0.675)	0.634<br>(95% CI: 0.604 to 0.665)
Incremental QALYs (adjusted)	0.002 (95% CI: -0.026 to 0.029)	
Total costs	£ 6173.83<br>(95% CI: £4231.94 to £8115.73)	£ 5610.79<br>(95% CI: £3652.69 to £7568.88)
Incremental costs (adjusted)	£987.37 (95% CI: -£1558.27 to £3533.02)	
**Cost-utility results**	**£20,000 cost-effectiveness threshold**	**£30,000 cost-effectiveness threshold**
Incremental net monetary benefit	-£961.27	-£942.45
Probability of NPWT being cost-effective	24.2%	25.5%

*Seemingly unrelated regressions

Figure 5: Cost-effectiveness plane (deaths excluded)

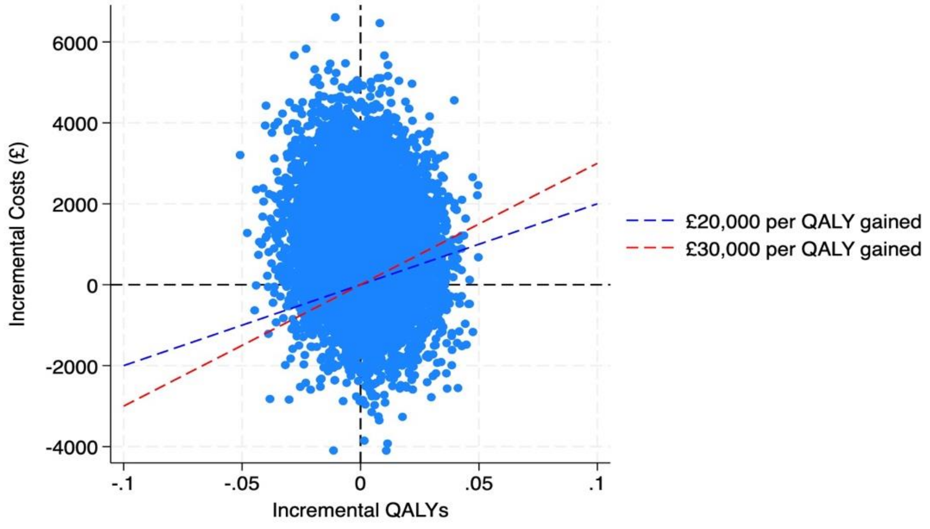

Figure 6: Cost-effectiveness acceptability curve (deaths excluded)

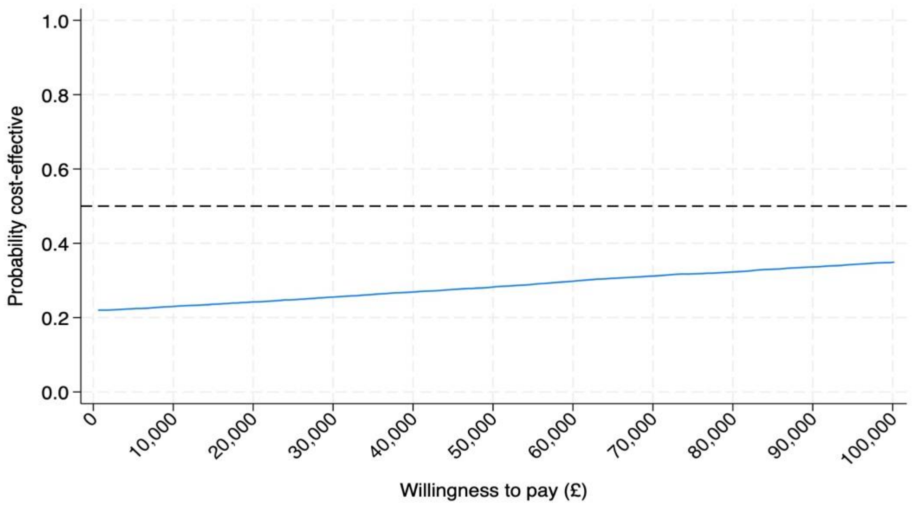

<u>*NPWT ever received (no intention to treat (ITT))*</u>

By accounting for treatment cross-over effects and participants who have ever received NPWT within the trial's follow-up period, the unadjusted mean per participant QALYs and mean per participant costs are 0.590 (95% CI: 0.557 to 0.622) and £ 6,404.83 (95% CI: £4,555.69 to £8,253.98) for the NPWT group and 0.555 (95% CI: 0.522 to 0.587) and £ 5,323.00 (95% CI: £3,470.69 to £ 7,175.29) for the usual care group. The mean difference-adjusted incremental QALYs and incremental costs suggest that NPWT increased QALYs by 0.024 (95% CI: --0.007 to 0.055) and increased costs by £1,260.83 (95% CI: --£1,383.88 to £3,905.54) over the 12-month study period, although these results were not statistically significant. The INMB is -£761.88 and -£516.18 at the £20,000 and £30,000 cost-effectiveness thresholds respectively. The probability

of cost-effectiveness of NPWT is 30.5% at the £20,000 threshold, and 37.3% at the £30,000 threshold. A cost-effectiveness plane and a CEAC present graphically these results (*Figure 7* and *Figure 8*).

Table xiv: Cost-utility analysis (NPWT ever received; no ITT)

**NPWT ever received (No intention to treat)**		
	**NPWT (n=333)**	**Usual care (n=353)**
Total QALYs	0.590 (95% CI: 0.557 to 0.622)	0.555 (95% CI: 0.522 to 0.587)
Incremental QALYs (adjusted)	0.024 (95% CI: --0.007 to 0.055)	
Total costs	£ 6404.83 (95% CI: £4555.69 to £ 8253.98)	£ 5323.00 (95% CI: £3470.69 to £ 7175.29)
Incremental costs (adjusted)	£1260.83 (95% CI: --£1383.88 to £3905.54)	
**Cost-utility results**	**£20,000 cost-effectiveness threshold**	**£30,000 cost-effectiveness threshold**
Incremental net monetary benefit	-£761.88	-£516.18
Probability of NPWT being cost-effective	30.5%	37.3%

*Seemingly unrelated regressions

Figure 7: Cost-effectiveness plane (NPWT ever received; no ITT)

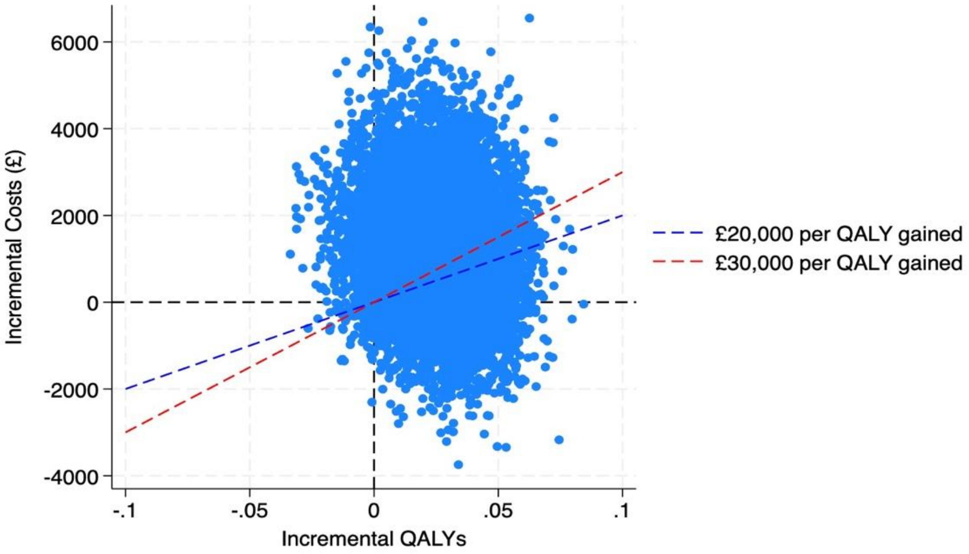

Figure 8: Cost-effectiveness acceptability curve (NPWT ever received; no ITT)

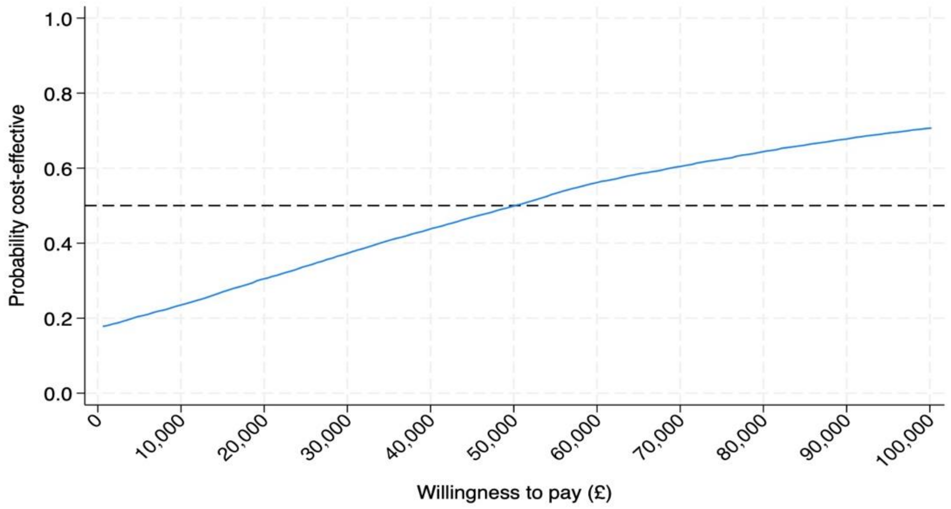

Supplement: Supplementary appendix [file mmc1.pdf]
